# Effect of the Solvent and Substituent on Tautomeric Preferences of Amine-Adenine Tautomers

**DOI:** 10.1021/acsomega.1c02118

**Published:** 2021-07-12

**Authors:** Anna Jezuita, Paweł Andrzej Wieczorkiewicz, Halina Szatylowicz, Tadeusz Marek Krygowski

**Affiliations:** † Faculty of Science and Technology, Jan Dlugosz University in Czestochowa, Al. Armii Krajowej 113/15, 42-200 Czestochowa, Poland; ‡ Faculty of Chemistry, Warsaw University of Technology, Noakowskiego 3, 00-664 Warsaw, Poland; § Department of Chemistry, Warsaw University, Pasteura 1, 02-093 Warsaw, Poland

## Abstract

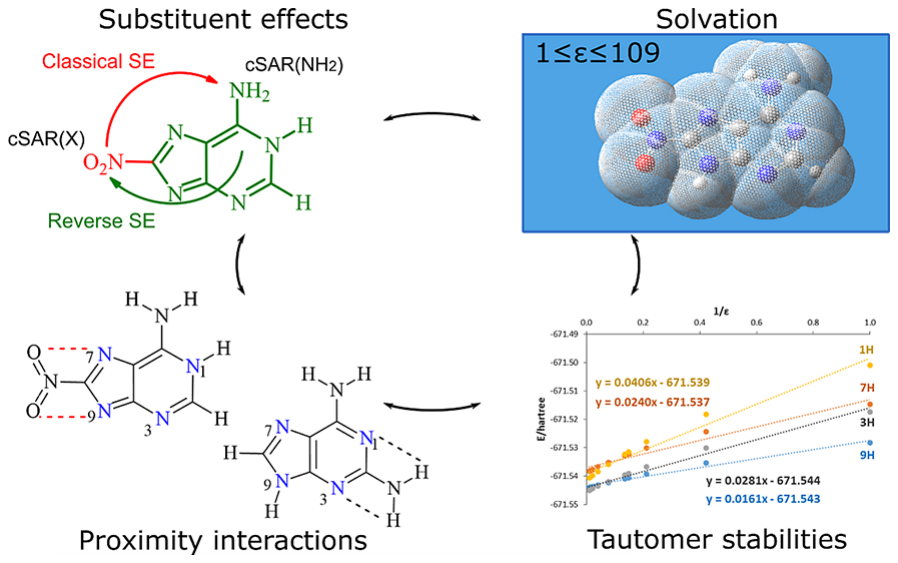

Adenine is one of the basic molecules of life; it is also an important building block in the synthesis of new pharmaceuticals, electrochemical (bio)sensors, or self-assembling molecular materials. Therefore, it is important to know the effects of the solvent and substituent on the electronic structure of adenine tautomers and their stability. The four most stable adenine amino tautomers (9H, 7H, 3H, and 1H), modified by substitution (C2– or C8−) of electron-withdrawing NO_2_ and electron-donating NH_2_ groups, are studied theoretically in the gas phase and in solvents of different polarities (1 ≤ ε < 109). Solvents have been modeled using the polarizable continuum model. Comparison of the stability of substituted adenine tautomers in various solvents shows that substitution can change tautomeric preferences with respect to the unsubstituted adenine. Moreover, C8 substitution results in slight energy differences between tautomers in polar solvents (<1 kcal/mol), which suggests that in aqueous solution, C8–X-substituted adenine systems may consist of a considerable amount of two tautomers—9H and 7H for X = NH_2_ and 3H and 9H for X = NO_2_. Furthermore, solvation enhances the effect of the nitro group; however, the enhancement strongly depends on the proximity effects. This enhancement for the NO_2_ group with two repulsive N···ON contacts can be threefold higher than that for the NO_2_ with one attractive NH···ON contact. The proximity effects are even more significant for the NH_2_ group, as the solvation may increase or decrease its electron-donating ability, depending on the type of proximity.

## Introduction

Adenine, as a part of the DNA/RNA helices,^1^ in a natural environment is subject to intermolecular interactions. They may lead to substantial changes in its electronic structure^2−4^ and, in consequence, to changes in its chemical/physicochemical/biochemical properties.^5,6^ Therefore, knowledge of changes in the electronic structure of adenine caused by both the solvent and well-defined factors (e.g., introduction of substituents) exhibiting specific electron donating/accepting properties is of fundamental importance. It should be emphasized that their influence on hydrogen bonds was most often studied in structurally modified Watson-Crick base pairs,^6−10^ whereas their influence on properties of nucleic acid bases is much less represented in the literature.^11,12^

The nature of the solvent has a significant impact on the properties and interactions of biomolecules; the effects of solvents may be a source of undesirable mutations in biological systems. Processes in real biological systems take place in both non-polar and polar environments.^13,14^ Recently, it has been shown that the polarity of the solvent changes the equilibrium constants of the double-proton transfer in G–C and A–T Watson–Crick base pairs.^15^ The solvent effect on hydrogen bonds in A–T pairs was also investigated.^16,17^ Experimental data on population of nucleic acid base tautomers in solution are rather rare and concern mostly uracil, thymine, and adenine. Concerning adenine, the structural parameters and energetic stability of all 23 tautomers were investigated computationally, taking into account both the different oxidation states and the aqueous environment.^18,19^ The three amino tautomers (N9H, N3H, and N7H) have been shown to be the most stable forms of adenine in the gas phase. Among them, the 9H tautomer is the most stable, which was confirmed by calculations in the gas phase and water^18−23^ and low-temperature matrix measurements.^24,25^ Detailed analysis of the electronic spectra of adenine and 2-aminoadenine suggests that the N7H and N3H tautomers, due to phototautomerization, can also be observed in aqueous solutions.^26−29^ The tautomeric equilibrium between the 9H and 7H forms of adenine was also studied in DMF and methanol by low-temperature ^1^H and ^13^C NMR spectroscopy,^30^ while fully ^15^N-labeled adenines were studied in DMSO-*d*_6_ using ^15^N NMR measurements.^31^ In the latter case, for adenine and 8-Br-adenine solutions, the spectra obtained suggested the existence of the N3H form in tautomeric mixtures, in addition to the well-reported N9H major tautomer and N7H minor tautomer. In addition, according to quantum chemical calculations for 8-Br-adenine in DMSO, the N3H tautomer was predicted to be slightly more stable than the N9H tautomer (by 0.4 kcal/mol), while the N7H isomer was less stable by 3.0 kcal/mol. Subsequent theoretical studies^11^ on the substitutional effect of halogen atoms (F, Cl, and Br) in the C8 position of adenine in the gas phase and in water showed that fluorine has the greatest influence on the stability of tautomers. Although its substituted N9H tautomer is the main form existing in the gas phase, the N3H and N9H tautomers are the major components of 8-F-adenine in aqueous solution; the obtained Boltzmann population ratios change in the order N3H > N9H > N1H > N7H (50.47, 35.32, 13.85, and 0.36%, respectively). Moreover, adenine can be part of the fluorescent nucleosides used to detect and study the structures and functions of nucleic acids, and their fluorescent properties are environmentally sensitive.^32^ Adenine is also part of ferrocenyl–nucleobase complexes exhibiting biological activity.^33^ Therefore, they are used to develop new pharmaceuticals, electrochemical (bio)sensors, or self-assembling molecular materials. Recently, the N7- and N9-isomers of ferrocenoylated adenine have been detected and isolated.^34^ In addition, interconversion was observed in a polar solvent (DMSO), at equilibrium with the more stable N9-isomer (88%). The N7/N9 isomerization reaction was not observed in less-polar and/or less-nucleophilic solvents (e.g., chloroform, acetone, and acetonitrile).

Thus, as mentioned above, it is very important to reveal the variability of system properties under the influence of external factors, simulated by substituents, especially in the case of systems with several tautomeric structures. In some special cases, such as interactions with cisplatin, binding to guanine or adenine leads to a fundamental mutation of the helix.^35,36^ As a result, cisplatin can be used as an important medicine in anti-cancer therapy. Therefore, in this work, substituents with strong electronic properties were selected—nitro and amine groups as typical electron-attracting (EA) and electron-donating (ED) substituents, respectively. The study of the influence of the nitro group on adenine is also important in the context of anticancer research. 8-Nitroadenine is one of the ROS-DNA (reactive oxygen species) adducts that are premutagenic and induce specific types of gene mutations,^37^ which alter gene expression and cause disturbances in the regulation of the cell cycle. 2-Aminoadenine (2,6-diaminopurine) is used in antileukemia treatment; it has antiviral and miRNA inhibition activity.^38−41^ Besides, adenine and its 2-amino derivative are used as ligands for purine riboswitches.^42^ In addition, it has recently been discovered that 2-aminoadenine is a drug candidate for the treatment of genetic diseases caused by UGA nonsense mutations.^43^ It has also been documented that the substituent at the C8 position of purine can greatly affect the rate of deoxyribosyl transfer to the base and the nature of the nucleoside formation.^44^ Furthermore, 8-chloroadenine was proved to be a useful biomarker for studying the role of reactive chlorine species during inflammatory processes.^45^ The oxidation at the C8 position of DNA bases has been found to be responsible for the tautomeric equilibrium between the normal and rare forms of DNA bases.^46^ Finally, 8-aryl adenine adducts are formed by the reaction of radical cation metabolites of carcinogenic polycyclic aromatic hydrocarbons (PAHs) with DNA.^47^ The substituents also influence base pairing interactions, for example, the amino group at the C8 position stabilizes Hoogsteen-type base pairing in triplex DNA.^48^ However, most of these studies do not provide information about the nucleobase at the molecular level. It is important to develop a systematic evaluation of substituted adenine derivatives. This may shine light on the chemical and biochemical properties of its nucleosides or nucleotides.

The solvent effect on processes influenced by substituent effects is usually considered using traditional substituent constants (SCs)^49,50^ or one of their many modifications.^51−54^ In general, the interpretation is realized using eq 1
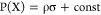
1where P(X) is the property in question and σ stands for the SC, whereas ρ is the reaction constant, describing the sensitivity of the process on the influence of the type of substituted species and the medium in which the measurements are carried out. If a given reaction series described by eq 1 is carried out in several solvents, the reaction constants ρ characterize quantitatively how the solvent influences the substituent effects observed in the process.^55,56^

It should be noted that the SCs are defined for a well-described reference reaction series, for example, the Hammett SCs are defined for the acid/base equilibria of meta- and para-substituted benzoic acids measured under standard conditions in water. However, these constants have not always worked sufficiently well, particularly for systems in which interactions between the substituents X and the reaction site Y differed significantly from those in the benzoic acid derivatives. Hence, the need to introduce other reference reaction series arises, resulting in numerous SC modifications.^51,52^ For a long time, SC values have been associated with the electronic structure of the substituent, namely, its ED or EA properties. In each type of SCs, we assume that the electronic structure of the substituent is fixed, but it varies depending on the type of the constants, that is, a reference system applied for their estimation. In other words, the electronic structure of a given substituent realized in various structural environments is described using differently defined SCs. The following question arises: how to describe changes in the electronic structure of a substituent as a result of its interactions with the substituted moiety and the environment? This problem may be solved using the quantum chemistry descriptor of the electronic structure of the substituent X, cSAR(X) (charge of the substituent active region).^57,58^ This is defined as a sum of atomic charges, *q*, of the substituent X and of the substituted carbon atom, C_ipso_, as presented by eq 2
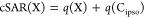
2

Unlike the atomic charges at substituents, *q*(X), the cSAR(X) values are nicely correlated with SCs for a series of mono-substituted^58^ and di-substituted benzene derivatives.^59^ It should be emphasized that cSAR(X) values estimated using various methods of estimating atomic charge assessments are well correlated.^60^

**Scheme 1:**
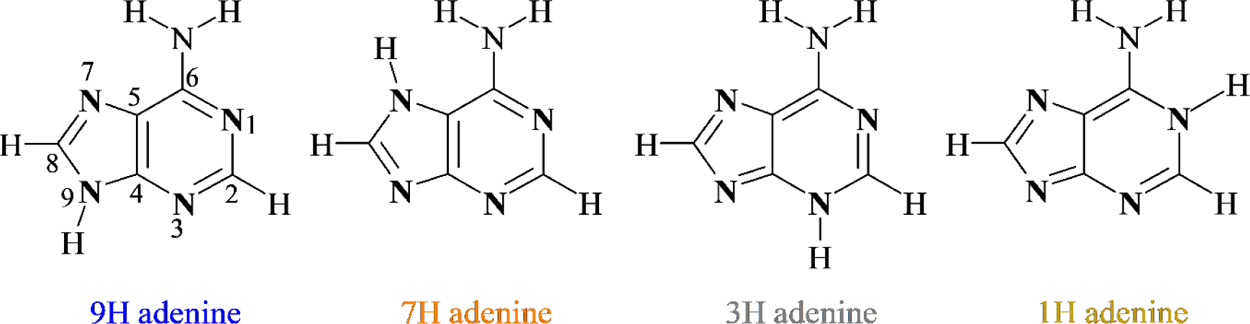
Structures and Numbering of Atoms of the Four Most Stable Amino Tautomers of Adenine

The idea of this paper is to investigate how the stability and electronic structure of adenine amino tautomers, described by the cSAR concept, depend on the solvent properties described by the so-called polarizable continuum model (PCM) model,^61^ where the solvent is considered as a continuous medium characterized by the dielectric constant ε. In our study, changes in medium properties are outspread from the gas phase (ε = 1.0) to the most polar, formamide (ε = 108.94). As objects of study, we have chosen derivatives of the four most stable amino adenine tautomers shown in Scheme 1.

Derivatives of the amino tautomers, substituted at the 2 or 8 position by one of two extremely different substituents—strongly ED NH_2_ (SC σ_p_ = −0.66) and EA NO_2_ groups (σ_p_ = 0.78, both values taken from ref (52)), may answer the above-posed questions.

It should be emphasized that in this paper, the substituent effect is mainly considered as the reverse substituent effect, that is, estimation of how the electronic properties of the X substituents in X–R–Y depend on R–Y, as previously presented for mono- and di-substituted benzene derivatives.^62−64^ In our case, we consider how the electronic properties of substituents depend on (i) the position of their attachment (C2 and C8 for X and C6 for the amino group in adenine) and (ii) the solvent in which estimations of cSAR(X) or cSAR(NH_2_) are realized.

## Computational Details

To study the influence of the solvent on the substituent effect, selected X substituents with different electronic properties (X = NO_2_, H, and NH_2_) were inserted into four adenine tautomers (Scheme 1) at the C8 or C2 position. For each studied system, optimization was carried out without any symmetry constraints (in the gas phase and in the solution) using the Gaussian09 program.^65^ According to the results of our previous research,^66^ the DFT-D method was used, namely, the B97D3 functional^67^ with Dunning’s aug-cc-pVDZ basis set.^68^ The choice of this computational level was due to its ability to characterize stacking interactions in adenine dimers; both energetic and geometric criteria were taken into account.^66^ The vibrational frequencies were calculated at the same level of theory to confirm that all calculated structures correspond to the minima on the potential energy surface. No imaginary frequencies were observed. To investigate the influence of selected solvents (see Table 1) on the electronic properties of substituents expressed by cSAR values, the PCM^61,69,70^ was used.

**Table 1:** Dielectric Constant, ε, Values for Studied Solvents

solvent/medium	acronym	ε
formamide	FA	108.94
water	H_2_O	78.36
DMSO		46.83
ethanol	Et-OH	24.85
pyridine	Py	12.98
THF		7.43
*o*-cresol	*o*-Cr	6.76
chloroform	ClF	4.71
toluene	Tol	2.37
gas phase	GP	1.00

The Hirshfeld method of atomic charge assessment^71^ was applied to calculate all cSAR values. The choice of this method was motivated by previous studies,^60^ in which Mulliken,^72^ AIM,^73^ Weinhold,^74^ and VDD^75^ charges were also taken into account.

## Results and Discussion

This section is divided into two parts. The first one is devoted to the solvent effect on the electronic structure of the substituent. The next subsection shows the influence of a solvent on the stability of substituted adenine tautomers. Selected solvents were modeled as a continuum of uniform dielectric constant, ε, using the PCM model,^61^ starting from ε = 1 (gas phase) up to ε = 108.94 (formamide; for all solvents, see Table 1).

### Impact of the Solvent on the Electronic Structure of a Substituent

**Figure 1:**
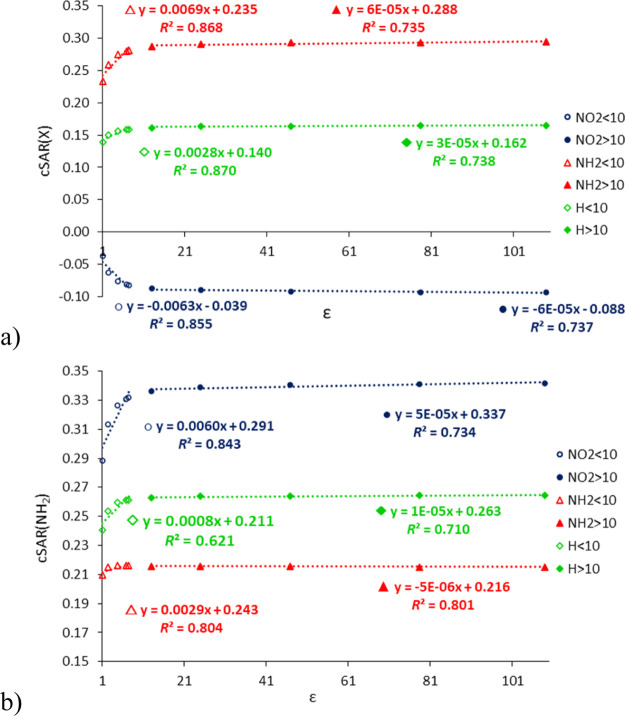
Dependences of cSAR(X) (a) and cSAR(NH_2_) (b) on ε for the C8–X-substituted 9H adenine tautomer (X = NO_2_, H, and NH_2_). The left and right linear regression equations correspond to two ε intervals, <10 and >10, with their determination coefficients (*R*^2^).

The influence of the solvent on the electronic structure of substituents can be studied using the cSAR concept. Obtained cSAR values for both the X substituent and the C6-attached NH_2_ group in the substituted adenine tautomers in the solvents under consideration are presented in Tables S1 and S2 (Supporting Information). It should be noted that as the ε value increases, monotonic changes in the cSAR of the substituents (X) and the reaction center (NH_2_) are observed. As shown in Figure 1, the dependences of cSAR on ε are curvilinear; however, separate linear relationships can be presented for two groups of solvents, with ε < 10 (ε_I_) and with ε > 10 (ε_II_). This separation provides valuable additional information on the relations of cSAR(X) versus ε. Figure 1 shows these relationships for the C8-substituted 9H adenine tautomer (data for all tautomers are presented in Table S3). Interestingly, in most cases, the increase in the dielectric constant is associated with an increase in cSAR(X) for X = NH_2_ (except for C8–NH_2_ systems for 3H and 1H tautomers and the C2–NH_2_ series for 7H tautomer) and a decrease for X = NO_2_. This is well documented by the positive and negative slope values of the linear regressions cSAR(X) on ε (Table S3). In other words, increasing the polarity of the solvent leads to an increase in the ED and EA ability of NH_2_ and NO_2_ substituents, respectively. In the case of the reaction site, the NH_2_ group at position C6, its ED properties increase for all analyzed structures, as shown in Figure 1b and Table S3.

Figure 1 shows that for media with ε < 10 (ε_I_), the changes in cSAR are much larger than for solvents with ε > 10 (ε_II_). This is clearly seen in the ranges of variability of cSAR values and the ratios ε_I_/ε_II_ presented in Table S4. Usually, these ratios are between 6 and 8, with only a few exceptions. It can also be shown by the percentages of the overall changes in cSAR(X) for ε < 10 and ε > 10 solvent groups (Table 2). These percentages for the former group are much higher, from 75.1 to 93.2%, than for the latter, so a further increase in the ε value above 10 has a very small effect on ED/EA properties of the substituents. The variability of cSAR(NH_2_) for the amino group at the C6 position is similar (Table S5). Clearly, the highest percentage variability in the ε > 10 group (24.9%) is for the C2–NH_2_ 7H system; however, the overall variability for this system is very small (0.011).

**Table 2:** Ranges of cSAR(X) Variation, Δ, and Percentages of Overall Variation for Media with ε < 10 and ε > 10 in C8–X- and C2–X-Substituted Adenine Tautomers

		cSAR(X)							
		C8–X				C2–X			
		ε < 10		ε > 10		ε < 10		ε > 10	
X		Δ	%	Δ	%	Δ	%	Δ	%
9H	NO_2_	0.045	87.5	0.006	12.5	0.062	89.9	0.009	10.1
	H	0.020	86.3	0.003	13.7	0.011	93.2	0.002	6.8
	NH_2_	0.049	87.3	0.007	12.7	0.001	91.7	0.000	8.3
7H	NO_2_	0.041	87.2	0.006	12.8	0.082	88.3	0.011	11.7
	H	0.019	87.4	0.003	12.6	0.016	84.5	0.003	15.5
	NH_2_	0.049	87.7	0.007	12.3	0.008	75.1	0.003	24.9
3H	NO_2_	0.095	86.7	0.015	13.3	0.015	92.6	0.001	7.4
	H	0.022	87.2	0.003	12.8	0.029	87.1	0.004	12.9
	NH_2_	0.024	92.7	0.002	7.3	0.064	87.5	0.009	12.5
1H	NO_2_	0.107	87.0	0.016	13.0	0.030	91.4	0.003	8.6
	H	0.032	85.9	0.005	14.1	0.028	86.5	0.004	13.5
	NH_2_	0.025	82.9	0.005	17.1	0.058	85.7	0.010	14.3

**Scheme 2:**
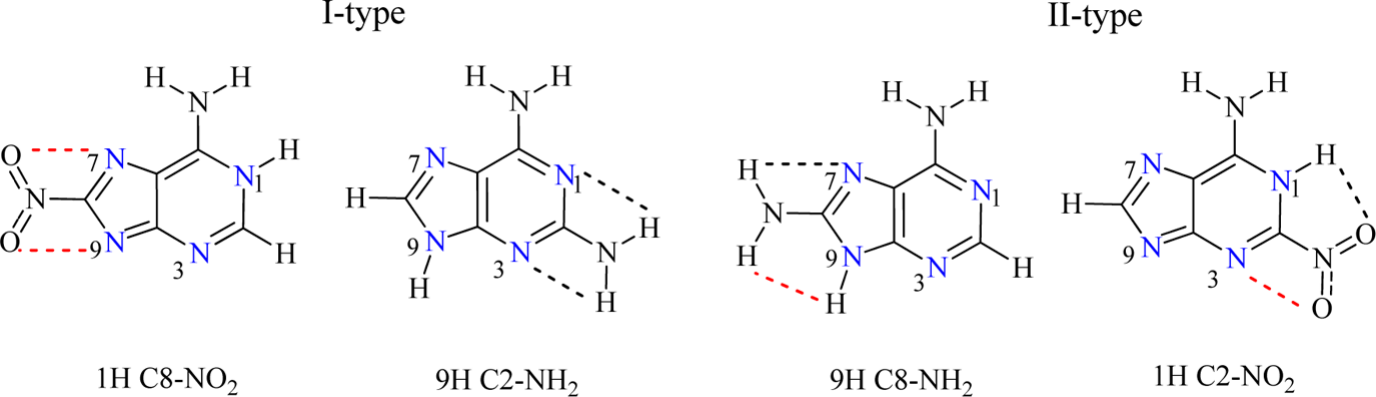
Possible Proximities of Substituents, with Two Neighboring Nitrogen Atoms with a Lone Pair in a Plane of the Ring (I-type) and with One Nitrogen Atom of This Kind and Another of the NH Type (II-type) Dashed lines denote through-space interactions with neighboring atoms, attractive black, repulsive red.

Looking at the ranges of cSAR(X) variability for particular X (NO_2_, H, and NH_2_) (Tables 3 and S1) in the studied solvents, a certain rule can be observed. When the nitro group interacts with neighboring pyridine-type nitrogen atoms (i.e., with lone pairs in the plane of the molecule, I-type proximity, Scheme 2), the average ranges of cSAR(NO_2_) variability at the C8 position for 1H and 3H tautomers and at the C2 position for 7H and 9H tautomers are 0.127 and 0.080, respectively. However, when this group interacts with pyridine- and NH-type nitrogen (II-type proximity, Scheme 2), the cSAR(NO_2_) values are 0.054 and 0.026, respectively. Thus, the effect of increasing solvent polarity on the range of cSAR(NO_2_) variability is 2.3–3.1 times greater for the I-type proximity than for the II-type one. This means that repulsive interactions in the first case are more sensitive to an increase in ε of the media than the partially repulsive (NO···N) and partially attracting (NO···HN) interactions in the latter.

The above-mentioned rule considering repulsive and attractive interactions is also realized in X = NH_2_ systems. However, a different character of this group, which in these cases is a hydrogen bond donor opposite to the NO_2_ group (a proton acceptor), results in a change in interactions occurring in I-type proximity. In contrast to the nitro group, the NH_2_ group in the I-type proximity experiences two attractive interactions (NH···N), while in the II-type proximity, one of them is repulsive (NH···HN), similar to NO_2_ (Scheme 2). As shown in the ratio column of Table 3, NH_2_ groups with II-type proximity are more sensitive to the solvent effect.

**Table 3:** cSAR(X) Ranges of Variation for Studied Solvents, Presented as the Mean Values for Two Tautomers Characterized by Similar Proximity; ε from 1.0 (Gas Phase) up to ε = 108.94 (Formamide)

X	position of substitution	Tautomers	type of proximity	mean range of cSAR(X) values	ratio (I-type/II-type)
NO_2_	C8	9H, 7H	II-type	0.054	2.35
		3H, 1H	I-type	0.127	
	C2	9H, 7H	I-type	0.080	3.08
		3H, 1H	II-type	0.026	
H	C8	9H, 7H	II-type	0.024	1.42
		3H, 1H	I-type	0.034	
	C2	9H, 7H	I-type	0.032	0.89
		3H, 1H	II-type	0.036	
NH_2_	C8	9H, 7H	II-type	0.062	0.48
		3H, 1H	I-type	0.030	
	C2	9H, 7H	I-type	0.007	0.09
		3H, 1H	II-type	0.078	

It should be emphasized that the above-discussed changes in cSAR values document the reverse substituent effect, that is, the impact of the substituted system on the substituent. In addition, these effects are of two types: (i) traditional resonance and inductive (field) interactions (effects) to a much greater degree (ii) proximity effects resulting from interactions with the ortho atoms/groups, in our case N or NH. The differences between the cSAR(X) values obtained in the gas phase and formamide are shown in the last column of Table S1. For unsubstituted adenine, these differences in cSAR(H) values (for C2–H and C8–H fragments) range from 0.014 to 0.041, while for substituents, they are as follows:

(a) for the NO_2_ group between 0.017 (3H, C2–NO_2_) and 0.135 (1H, C8–NO_2_), with the mean difference of 0.074, and
(b) for the NH_2_ group between 0.001 (9H, C2–NH_2_) and 0.080 (3H, C2–NH_2_), with the mean difference of 0.044.

Therefore, the largest differences refer to the C8-nitro and C2-amino derivatives in 1H and 3H tautomers, respectively. The mean range of cSAR(X) changes (including all substituents; X = NO_2_, H, and NH_2_) for all four C2- and C8-substituted adenine tautomers in the gas phase is 0.264. Thus, changes in cSAR(NO_2_) constitute 0.074/0.264 part of the overall variability of cSAR(X) (considering the scale between NH_2_ and NO_2_), that is, 28%, and for cSAR(NH_2_) are 0.044/0.264, that is, 17%. Consequently, the effects of changes in the cSAR(X) value due to an environment change, from the gas phase to formamide, can account for about a quarter of the total variation in the cSAR(X) scale in the gas phase. Moreover, the total variability of the cSAR(X) scale also increases with an increase in the solvent dielectric constant. In the case of formamide, it is about 40% higher than that determined for the gas phase, due to stabilization of both a negative charge on the EA nitro group and a positive charge on the ED amino group.

The situation is different when we consider the classical substituent effect, that is, how the X substituents affect the electronic properties of the reaction site Y—the amino group in the C6 position of adenine. It is clearly seen that more pronounced changes in cSAR(NH_2_) values (Tables 4 and S2) are observed for the 7H and 1H tautomers, when this amino group is adjacent to the endo-NH group (i.e., II-type proximity). This indicates that the solvent affects the strength of the substituent effect but does not change the nature of interactions of the substituents with the amino group. In general, the increase in the polarity of the solvent causes the enhancement of intramolecular interactions by the nitro group and weakening by the amino group (as a substituent). The greatest strengthening and weakening of the classical substituent effects, compared to X = H, are observed for the C8-substituted derivatives of the 9H tautomer (Table 4).

**Table 4:** cSAR(NH_2_) Ranges of Variation, Δ_Y,I(II)_ (Y = NH_2_ Group at the C6 Position of Adenine, Subscripts 1 and 2 Indicate Its Proximity Type), and Their Ratio with Respect to X = H Adenine Tautomers for Studied Solvents; ε from 1.0 (Gas Phase) up to 108.94 (Formamide)

				range of cSAR(NH_2_) Δ_Y,I_	range of cSAR(NH_2_) Δ_Y,II_	Δ_Y,I_/Δ_H,I_	Δ_Y,II_/Δ_H,II_
X	position of substitution	tautomers	type of proximity for X	9H or 3H	7H or 1H	9H or 3H	7H or 1H
NO_2_	C8	9H, 7H	II-type	0.053	0.105	2.21	1.31
		3H, 1H	I-type	0.062	0.132	1.72	1.13
	C2	9H, 7H	I-type	0.040	0.102	1.67	1.28
		3H, 1H	II-type	0.052	0.112	1.44	0.96
H		9H, 7H		0.024	0.080		
		3H, 1H		0.036	0.117		
NH_2_	C8	9H, 7H	II-type	0.007	0.056	0.29	0.70
		3H, 1H	I-type	0.017	0.104	0.47	0.89
	C2	9H, 7H	I-type	0.016	0.072	0.67	0.90
		3H, 1H	II-type	0.027	0.111	0.75	0.95

According to Coulomb’s law, apart from the distance between the charges, the dielectric permittivity, in the form 1/ε, determines the interaction forces between charges. It should be stressed that about 90% of the interaction force is realized for the ε range between 1 and 10 (1/ε between 0.1 and 1).

**Figure 2:**
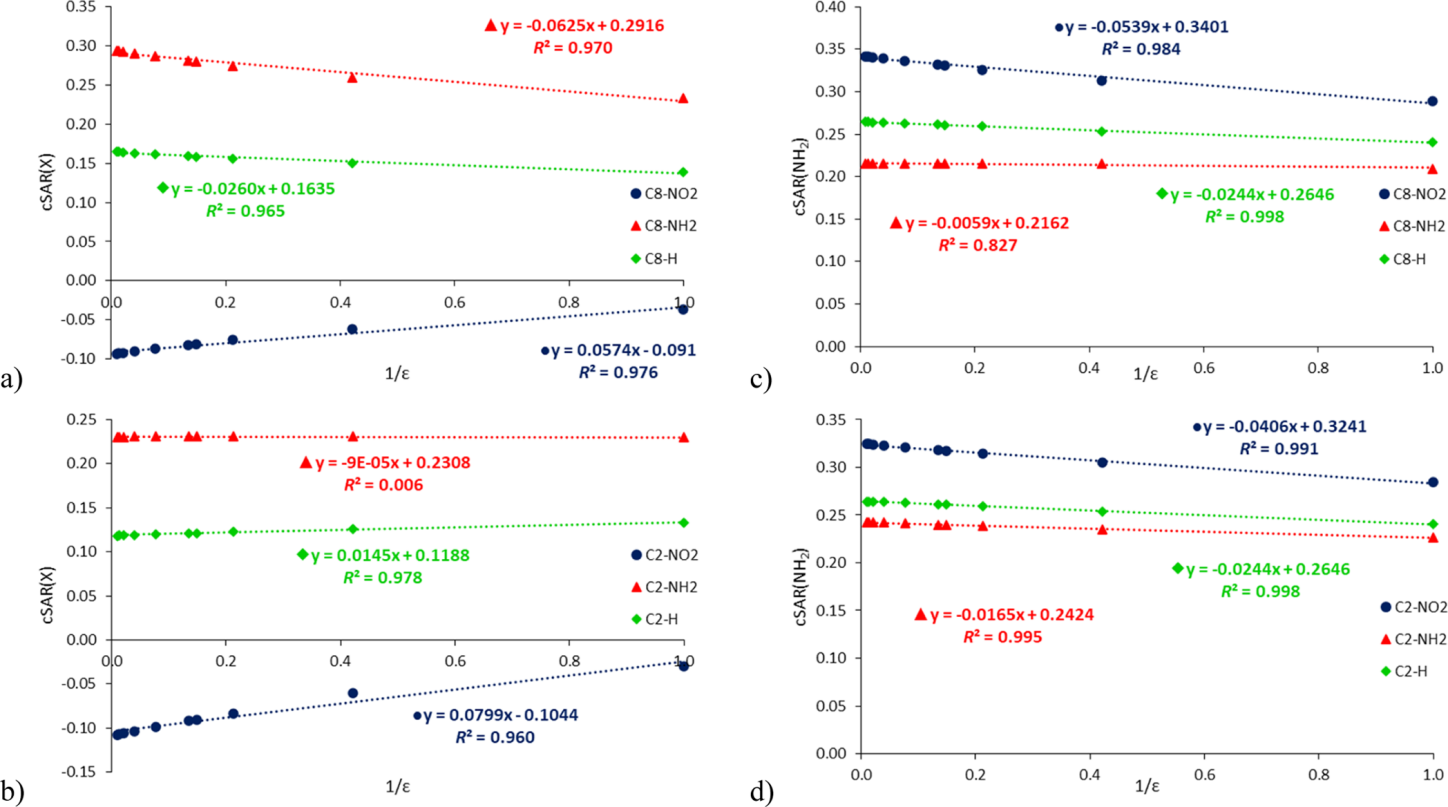
Dependences of cSAR(X) (a,b) and cSAR(NH_2_) (c,d) on the reciprocal of solvent permittivity 1/ε for C8–X and C2–X substitution of the 9H adenine tautomer.

Various substituted adenine tautomers are dipolar in nature. Thus, according to Coulomb’s law of interacting electric charges, it seems reasonable to plot the changes in cSAR(X) or cSAR(NH_2_) against the dielectric permittivity reciprocity, 1/ε. Contrary to the relations in Figure 1, these relations are linear, as shown for 9H adenine tautomer derivatives in Figure 2. Similar relations for other tautomers are presented in Figures S1–S6. The obtained slopes of the linear equations cSAR(X) or cSAR(NH_2_) versus 1/ε and the determination coefficients for all systems are presented in Table S6. It should be emphasized that these data are generally very well correlated, except in only a few cases when the solvent hardly changes the electronic structure of the substituent (e.g., for X = NH_2_ in the C2-substituted 9H tautomer).

The slopes of these regressions provide similar information on the dependence of cSAR(X) and cSAR(NH_2_) on the solvent dielectric permittivity in various adenine systems as the data presented in Tables 3 and 4. The signs of the slopes are additional important information. They show the already discussed differences in the influence of the solvent polarity on the electronic structure of the substituent and the reaction site. For the NO_2_ group, the slopes of these linear equations are always positive; the EA ability of this group increases with the solvent dielectric permittivity. In the case of cSAR(X) of the NH_2_ group, the sign of the slopes is positive for I-type proximity and negative for II-type proximity. It follows that for the I-type proximity, that is, when the *endo*-NH group is in a different ring of adenine from the substituent, a decrease in cSAR(X) with the increase in the solvent polarity is observed, which indicates the weakening of its ED ability. On the other hand, II-type groups, with the *endo*-NH group in the same ring, exhibit an increase in cSAR(X), which corresponds to an enhancement of intramolecular interactions. Interestingly, the C6–NH_2_ group does not follow this rule; its cSAR(NH_2_) increases in all cases, regardless of the proximity type.

Changes in cSAR are also connected with the changes in CN bond lengths, collected in Table 5 (for bond lengths, see Table S7). When solvation causes an increase in the ED ability of the group, as for X = NH_2_ in the II-type proximity and all C6–NH_2_ groups and in all cases for X = NO_2_, the bonds are shorter in a polar environment. The opposite is true when the ED ability decreases (as only for X = NH_2_ in the I-type proximity), that is, lengthening of CN bonds is observed. The extent to which bonds are shortened depends on the proximity of the X or NH_2_ group. For X = NO_2_, the shortening of the C–N bond is larger by 0.008 Å on average in the cases when the NO_2_ group has I-type proximity. For C6–NH_2_, bonds are shortened more when the C6 amino group has II-type proximity, that is, for 1H and 7H tautomers. An average shortening of this bond for the I-type proximity is almost negligible (0.005 Å), whereas for the II-type one, it is more than four times larger: 0.023 Å. In summary, the weakening of repulsive electrostatic interactions between N···O and NH···HN due to the increasing ε of the environment seems to be the main factor governing the changes in CN bonds lengths and the changes in EA/ED properties of substituents due to solvation, as quantified by cSAR. This is an interesting example of how differently solvation can affect the electronic and geometric properties of substituents due to the proximity effects.

**Table 5:** Differences (Δ) of C^X^–N and C^6^–N Bond Lengths (in Å) between Formamide (ε = 108.94) and the Gas Phase (ε = 1)^a^

		X = NH_2_		X = NO_2_		
		ΔC^X^–N	ΔC^6^–N	ΔC^X^–N	ΔC^6^–N	type of proximity X
9H	C2	0.0011	–0.0025	–0.0099	–0.0065	I
7H		0.0035	–0.0188	*0.0122*	–0.0253	I
3H		–0.0199	–0.0042	–0.0037	–0.0075	II
1H		–0.0204	–0.0274	–0.0074	–0.0198	II
9H	C8	–0.0141	–0.0003	–0.0118	–0.0079	II
7H		–0.0148	–0.0145	–0.0121	–0.0243	II
3H		0.0031	–0.0035	–0.0206	–0.0086	I
1H		0.0085	–0.0252	–0.0215	–0.0248	I

a In the case of 7H C2–NO_2_, positive change (marked in italics) is associated with a rotation of the NO_2_ group and was not considered in average values discussed above.

**Figure 3:**
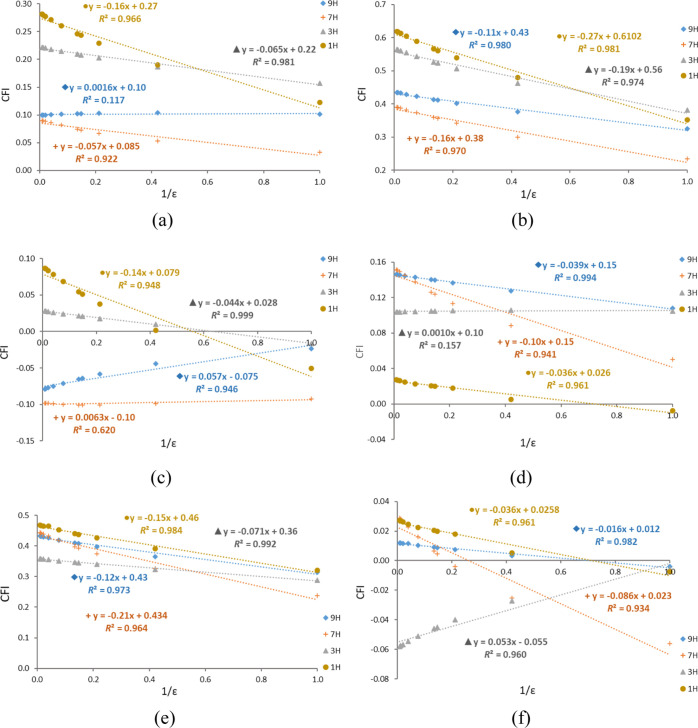
Dependences of the CFI on the reciprocal of solvent permittivity 1/ε for C8–X, X = H (a), X = NO_2_ (b), and X = NH_2_ (c) and C2–X, X = H (d), X = NO_2_ (e), and X = NH_2_ (f) substituted adenine tautomers.

Undoubtedly, the electronic structure of substituents is associated with their interactions with the C6 amino group and proximity effects. These can be described by the charge flow index, CFI, [CFI = cSAR(NH_2_)–cSAR(X)]. Similar to the cSAR(X) and cSAR(NH_2_) values, the CFI values depend on the dielectric permittivity, as shown in Figure 3.

Due to the stabilization of charge on substituents, for C2– and C8–NO_2_-substituted adenine tautomers, the CFI linear regressions have negative slopes, that is, CFI values decrease with an increase in 1/ε. This means that as the “ionizing power” of the medium increases, the charge flow between the amino and nitro groups increases. The variability in the slopes is similar in both cases: between −0.07 and −0.21 for C2–NO_2_ compared to −0.11 and −0.27 for C8–NO_2_. Moreover, in all cases, determination coefficients are high (*R*^2^ > 0.965), indicating a high level of fulfillment of the similarity model. This is not the case for C2– and C8–NH_2_-substituted systems. The precision of the regression lines is slightly lower, but differentiation between the slope values is very significant: between −0.09 and 0.05 for C2–NH_2_ and between −0.14 and 0.06 for C8–NH_2_-substituted systems.

High negative slopes are found when the endo-NH group is near the NH_2_ group at the C6 position of adenine (II-type proximity) and the second amino group (at the C2 or C8 position) has I-type proximity. In this case, as mentioned earlier, the C6 amino group experiences better stabilization of charge, while the charge on the second amino group is weakly stabilized. Thus, the solvent has a much greater effect on the charge of the C6 amino group than the other.

High positive slopes, as in the cases of 9H C8–NH_2_ and 3H C2–NH_2_, represent the opposite situation; the amino group at C8 and C2, respectively, has N and NH proximity (II-type), while the C6 amino group has two pyridine-type N atoms in its proximity (I-type). Again, the electronic properties of one amino group are much more affected by the solvent, but according to the CFI formula, these changes have an opposite sign.

The cSAR relationships discussed above show the effect of the solvent on a local charge distribution at the substituents and the reaction site. The global charge distribution in the molecule can be characterized by the dipole moment, μ. Dependences of the molecular dipole moment on the reciprocal of solvent permittivity are shown in Figure S7, while graphical representations of molecular dipole moments in the gas phase and formamide are shown in Figure S8.

For NO_2_-substituted adenine tautomers, the slopes of μ versus 1/ε dependences are consistent with the slopes of CFI versus 1/ε relations. This is understandable since the changes in μ result from changes in the molecular charge distribution that mainly occur at the substituents. The increase in the CFI of NO_2_-substituted adenines evidences the higher difference of charge at the NO_2_ and NH_2_ groups, and thus, an increase in the dipole moment is observed. When X = NH_2_, for both C2–X and C8–X substitution, 1H shows the best stabilization of the molecular dipole moment. This results from the fact that in the 1H tautomers, the C6–NH_2_ group exhibits the highest variability of cSAR due to solvation (0.104 and 0.111 for C8–X and C2–X, respectively; see Table S2), that is, solvation causes the highest accumulation of positive charge on this group.

### Effect of the Solvent on Tautomer Stability and Solvation Energy

**Figure 4:**
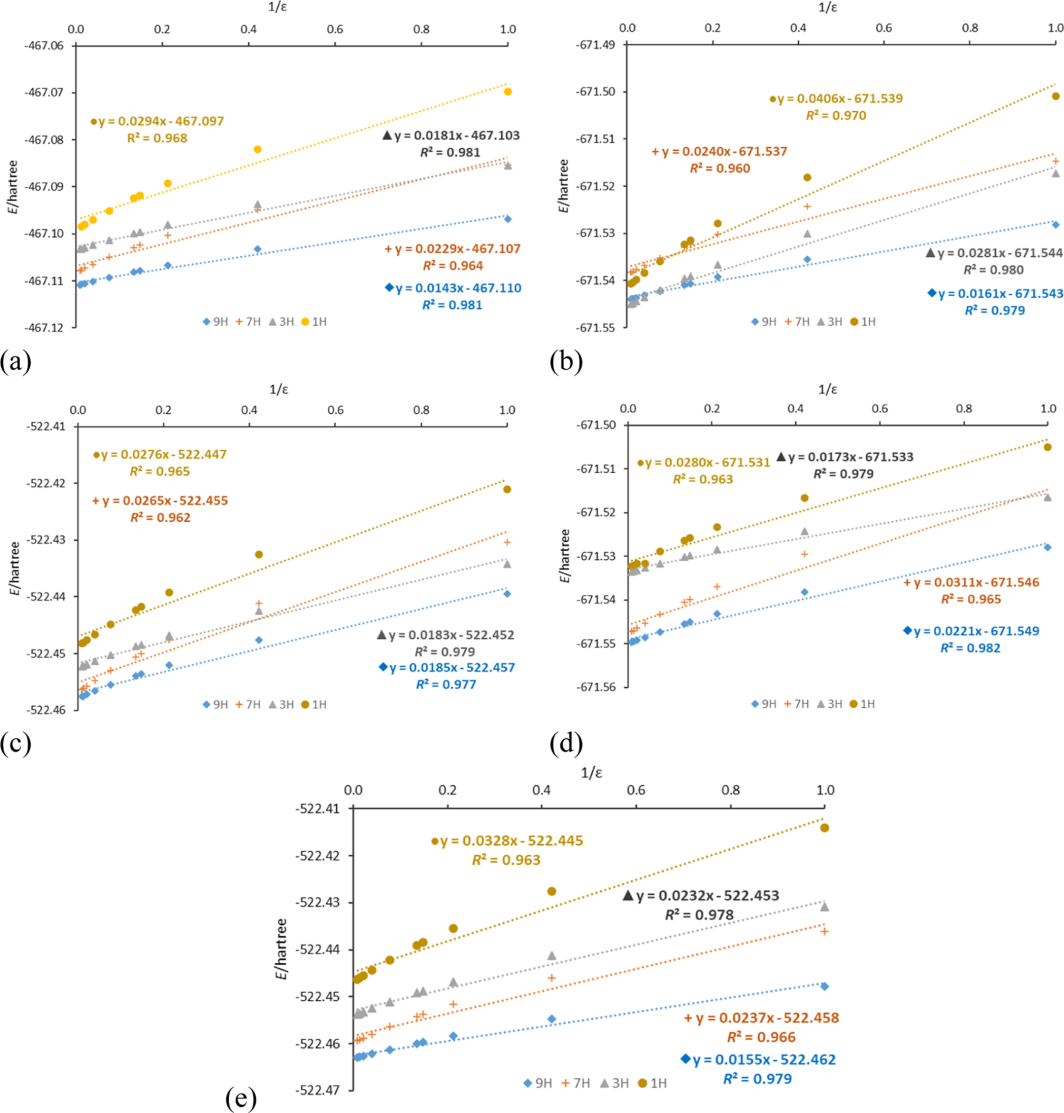
Dependences of total electronic energy, *E* (in hartrees), on 1/ε in unsubstituted (a) and C8–NO_2_- (b), C8–NH_2_- (c), C2–NO_2_- (d), and C2–NH_2_ (e)-substituted adenine tautomers.

As shown in a recent study on the stability of substituted (C2–X and C8–X) adenine amino tautomers in the gas phase,^76^ substituted 9H tautomers are the most stable, whereas 1H are the least stable. As for the 7H and 3H systems, they display stability between 9H and 1H, but their mutual stability varies depending on the substituent and its position. The presence of a solvent can significantly change the stability of substituted adenine tautomers, as shown in Table 6 and Figure 4. In addition, an increase in solvent polarity leads to a reduction in energy differences between the most- and least-stable tautomers in all analyzed cases; this was previously found also for the 8-halogen adenine derivatives.^11,31^ A valuable illustration of the solvent effect on stability is provided by dependences of total electronic energy on 1/ε, presented in Figure 4. These relations present not only the stability sequence of 1H, 3H, 7H, and 9H tautomers in each studied solvent but also to what extent the increase in polarity of the solvent stabilizes each tautomer, as shown by the slopes of linear fit equations. Values of these slopes follow the same sequence as the slopes of μ versus 1/ε relations (Figure S7).

**Table 6:** Relative Energies (Relative to the 9H Tautomer), *E*_rel_, Obtained for 7H, 3H, and 1H Adenine Tautomers Substituted at the C8 and C2 Positions^a^

		E_rel_/kcal•mol^–1^									
		GP (1.00)	Tol (2.37)	ClF (4.71)	*o*-Cr (6.76)	THF (7.43)	Py (12.98)	Et–OH (24.85)	DMSO (46.83)	H_2_O (78.36)	FA (108.94)
H	9H	0.00	0.00	0.00	0.00	0.00	0.00	0.00	0.00	0.00	0.00
	7H	7.18	5.31	3.98	3.42	3.30	2.70	2.27	2.03	1.91	1.86
	3H	7.09	6.00	5.43	5.22	5.18	4.98	4.84	4.77	4.74	4.72
	1H	16.94	13.32	10.97	10.05	9.85	8.90	8.23	7.86	7.69	7.62
C8–NO_2_	9H	0.00	0.00	0.00	0.00	0.00	0.00	0.00	0.00	0.00	0.00
	7H	8.50	6.96	5.70	5.15	5.03	4.43	3.99	3.75	3.64	3.59
	3H	6.81	3.38	1.60	0.96	0.82	0.19	–0.24	–0.47	–0.58	–0.62
	1H	17.16	10.85	7.11	5.70	5.39	3.96	2.95	2.41	2.16	2.05
C8–NH_2_	9H	0.00	0.00	0.00	0.00	0.00	0.00	0.00	0.00	0.00	0.00
	7H	5.70	4.09	2.80	2.27	2.15	1.59	1.18	0.95	0.85	0.80
	3H	3.77	3.24	3.27	3.29	3.30	3.32	3.33	3.34	3.35	3.35
	1H	11.50	9.46	8.04	7.46	7.33	6.72	6.28	6.04	5.92	5.87
C2–NO_2_	9H	0.00	0.00	0.00	0.00	0.00	0.00	0.00	0.00	0.00	0.00
	7H	7.09	5.39	3.90	3.29	3.16	2.51	2.01	1.74	1.61	1.55
	3H	7.17	8.68	9.31	9.55	9.60	9.83	9.99	10.07	10.11	10.13
	1H	14.39	13.52	12.52	12.10	12.01	11.55	10.60	11.01	10.92	10.88
C2–NH_2_	9H	0.00	0.00	0.00	0.00	0.00	0.00	0.00	0.00	0.00	0.00
	7H	7.32	5.50	4.22	3.70	3.58	3.04	2.63	2.41	2.31	2.26
	3H	10.61	8.47	7.30	6.88	6.79	6.37	6.08	5.94	5.87	5.84
	1H	21.15	17.07	14.38	13.32	13.09	11.99	10.60	10.77	10.57	10.48

a In brackets are dielectric constants of solvents.

In all but one case, 9H remains the most stable tautomer. The difference can be noticed for the C8–NO_2_-substituted adenine tautomers (Figure 4b), where the 3H tautomer is more sensitive to the solvation effect than the 9H (*E* vs 1/ε slopes 0.0278 and 0.0161, respectively). Moreover, the 3H tautomer is more stable than 9H in ethanol and more polar solvents. In the case of the C8–Br derivative, the presence of this tautomer in DMSO solution has already been confirmed experimentally.^31^ Higher sensitivity to the solvation effect results from changes in the molecular dipole moment of 3H (Figure S7b), as shown by the slopes of μ versus 1/ε relations: −1.276 for 9H and −4.187 for 3H. In the gas phase and less-polar solvents, the 1H tautomer is the least-stable amino tautomer in all substitution cases. However, in general, it exhibits the highest sensitivity to the solvent. In addition, for C8–NO_2_-substituted systems in solvents with ε > 10, better stability of the 1H than the 7H tautomer is observed. To easily assess the stability differences between the tautomers for particular substitutions and in each solvent, relative values of energy, *E*_rel_, (in kcal/mol) are presented in Table 6. Their values clearly show that the solvent can significantly reduce the relative energies, especially for the 7H tautomer (for X = H from 7.18 in the gas phase to 1.91 kcal/mol in water, in agreement with previous studies).^12,18^ Moreover, the substituent, that is, intramolecular interactions, can change tautomeric preferences, as shown above.

For unsubstituted adenine (X = H), a monotonic change in stability is observed in the following order: 9H, 7H, 3H, and 1H. *E*_rel_ decreases in all cases when ε increases (except for 9H, which is a reference system).

In the case of X = NO_2_, the situation is more complex. For C8–NO_2_ substitution, all other tautomers are better stabilized than 9H, that is, *E*_rel_ decreases with an increase in ε. However, the sequence of stability depends on the considered range of dielectric permittivity. For less-polar solvents, ε < 10, stability decreases in the following order: 9H, 3H, 7H, and 1H. In pyridine, the sequence is 9H, 3H, 1H, and 7H, while for ethanol and more polar solvents, the order is 3H, 9H, 1H, and 7H. The simpler situation is for C2–NO_2_, where a monotonic change in stability is observed in the following order: 9H, 7H, 3H, and 1H. For 3H, *E*_rel_ increases with increasing ε; hence, it is stabilized worse than for 9H, which is also shown by the slopes of *E* versus 1/ε relations (Figure 4d): 0.0173 for 3H and 0.0221 for 9H.

In amino-substituted systems, the stability sequences are less complex. In the case of C8–NH_2_ in the gas phase and toluene, the order is 9H, 3H, 7H, and 1H, while in solvents with ε > 4, the order is 9H, 7H, 3H, and 1H. *E*_rel_ is almost constant for the 3H substituted tautomer, as it is stabilized to a similar extent as 9H (*E* vs 1/ε slopes are alike; see Figure 4c). In the case of C2–NH_2_, a monotonic change in stability is observed in the following order: 9H, 7H, 3H, and 1H. *E*_rel_ decreases when ε increases.

An interesting insight is provided by the solvation energies in different solvents (Figure S9). For unsubstituted adenine, the energy of solvation, *E*_solv_ = *E*_in solvent_ − *E*_GP_, increases (as absolute values) in the sequence 9H, 3H, 7H, and 1H regardless of the type of solvent. In the 7H and 1H tautomers, the *endo*-NH group is close to the C6 amino group, whereas the other two tautomers contain a pyridine-type nitrogen at these positions, which to some extent may lower their solvation energy due to the small dipole moment of 3H and 9H tautomers (Figure S7).

When there is an additional substituent (NO_2_ and NH_2_) in the adenine molecule, relations between solvation energies of tautomers can change. In the case of C8–NO_2_-substituted adenine derivatives, the solvation energies for particular tautomers increase (as absolute values) from 9H, 7H, and 3H to 1H. One should note that 1H and 3H tautomers contain a negatively charged NO_2_ group in a 5-membered ring and a positively charged NH_2_ and *endo*-NH in a 6-membered ring. This results in a more dipolar electronic structure than in the other two tautomers, in which the NH group is adjacent to the NO_2_ group. In the case of C8–NH_2_, 1H and 7H tautomers are better solvated. This is due to the contribution of positively charged NH_2_ and NH groups in one part of a molecule, whereas the other part contains negatively charged N3 and N9 atoms with lone pairs in a plane of the rings.

A somewhat similar situation occurs with the C2–X-substituted adenine tautomers. Nitro derivatives are more strongly solvated in the case of 1H and 7H tautomers, where again, the *endo*-NH group in the 1 or 7 position is close to the C6–NH_2_ group, increasing the overall dipole moment. Hence, a stronger solvation is observed. In C2–NH_2_ derivatives, the 1H tautomer is solvated significantly stronger than 3H and 7H, as it consists of three positively charged groups, *endo*-NH and two NH_2_, in one part of the molecule. The smallest *E*_solv_ values are observed for 9H, where the contribution to the dipole moment from the C6–NH_2_ group is neutralized by the local dipole moment at N9–H.

## Conclusions

The aim of this study was to show how nucleophilic/electrophilic factors, simulated by electron attracting/donating substituents, and solvents influence the stability and electronic structure of substituted adenines. For this purpose, nitro and amino groups were selected as substituents (attached to the C8 or C2 position of adenine) along with a wide range of solvent properties (from the gas phase, ε = 1, to formamide, ε = 108.94). The influence of the solvent on the stability and the local and global electronic structure of the substituted 9H, 7H, 3H, and 1H adenine tautomers was investigated using density functional theory and the PCM solvent model. The use of the cSAR (charge of the substituent active region) model made it possible to compare changes in the local electronic structure, that is, the substituent effects (classical and reverse) in 10 environments.

The solvent effect can significantly change the stability of substituted adenine tautomers. The increase in solvent polarity leads to a decrease in energy differences between the most- and the least-stable tautomers in all analyzed cases. Moreover, both the solvent and substitution can change tautomeric preferences. For adenine, the gas phase stability sequence of 9H, 3H, 7H, and 1H already changes in toluene (ε = 2.37) to 9H, 7H, 3H, and 1H. In the case of C8–NH_2_ systems, the same change takes place in chloroform. However, among the C8–NO_2_ adenine tautomers, the 3H tautomer is the most stable in ethanol (and more polar solvents), followed by 9H, 1H, and 7H. In addition, for the C8–X-substituted systems, slight energy differences (<1 kcal/mol) are found between the tautomers in polar solvents. This suggests that a significant amount of two tautomers can coexist in DMSO and more polar solvents—9H and 7H for X = NH_2_, while 3H and 9H for X = NO_2_.

Monotonic changes in the cSAR values of the substituents (X) and the reaction center (NH_2_) and the dipole moment, μ, with an increase in the value of ε are observed. However, these changes are much greater in solvents with ε < 10 than ε > 10. Moreover, the application of Coulomb’s law allows these relationships to be linearized for a whole range of solvents. Generally, the resulting linear equations (as a function of the relative permittivity reciprocity, 1/ε) are very well correlated.

Changes in cSAR due to the influence of the solvent depend on the substituent, its attachment position, and the type of the tautomer. Moreover, changes in cSAR(X) are strongly influenced by proximity effects. The reverse substituent effect (i.e., the impact of the substituted system on the substituent) for the NO_2_ group with two repulsive NO···N contacts (I-type proximity) can be three times greater than that for the NO_2_ with one attractive NO···HN contact (II-type proximity). The opposite is true for X = NH_2_; the II-type proximity interactions (NH···N and NH···HN) are more sensitive to the solvent effect than the I-type (two attracting NH···N).

The obtained cSAR(X) versus 1/ε linear relations show that for X = NH_2_, the signs of the slopes are positive for systems with I-type proximity and negative for II-type proximity. Thus, in the first case (C8–NH_2_ in 1H and 3H tautomers and C2–NH_2_ in 7H and 9H systems), a decrease in cSAR(X) is observed with increasing solvent polarity. This indicates a weakening of the NH_2_ ED ability. In contrast, II-type groups exhibit an increase in cSAR(X), which corresponds to an enhancement of intramolecular interactions. However, changes in the electronic structure of the C6–NH_2_ group do not follow this rule. Its cSAR(NH_2_) increases in all cases, regardless of the proximity type. For the nitro group, the slopes are always positive, so its EA ability increases with the solvent dielectric permittivity. Moreover, changes in cSAR are also associated with changes in CN bond length.

## Supporting Information Available

cSAR(X) and cSAR(NH_2_) values for C8–X and C2–X substitution in different solvents (S1 and S2); slopes of linear equations and determination coefficients for dependences of cSAR(X) and cSAR(NH_2_) on ε (S3); ranges of cSAR(X) variability and their ratios (ε_I_/ε_II_) for media with ε < 10 (ε_I_) and ε > 10 (ε_II_) for C8–X and C2–X-substituted adenine tautomers (S4); ranges of cSAR(NH_2_) variability and their percentages for ε < 10 and ε > 10 series in C8–X and C2–X adenine tautomers (S5); slopes of linear equations and determination coefficients for dependences of cSAR(X) and cSAR(NH_2_) on 1/ε for C8–X- and C2–X-substituted adenine tautomers (S6); C–N Bond lengths in the gas phase and formamide, their difference and type of proximity (S7); dependences of cSAR(X) on 1/ε for C8–X- and C2–X-substituted 7H, 3H, and 1H tautomers; dependences of cSAR(NH_2_) on 1/ε for C8–X- and C2–X-substituted 7H, 3H, and 1H tautomers; dependences of the molecular dipole moment on the reciprocal of solvent permittivity, 1/ε, for unsubstituted and C8–NO_2_-, C8–NH_2_-, C2–NO_2_-, and C2–NH_2_-substituted adenine amino tautomers; graphical representations of molecular dipole moments in the gas phase and formamide; and solvation energies of unsubstituted and C8–NO_2_-, C8–NH_2_-, C2–NO_2_-, and C2–NH_2_-substituted adenine tautomers in studied solvents (PDF)

## Dedication

Dedicated to our friend, Professor Adam Proń, on the occasion of his 70th birthday.
