# Supplementary material for: Effect of the Solvent and Substituent on Tautomeric Preferences of Amine-Adenine Tautomers
Source: ACS Omega. 2021 Jul 12;6(29):18890–903. doi: 10.1021/acsomega.1c02118 (PMC8320138; doi:10.1021/acsomega.1c02118)
Supplement: Supplementary file 1 — ao1c02118_si_001.pdf [file ao1c02118_si_001.pdf]

SUPPORTING INFORMATION (SI)

Effect of the Solvent and Substituent on Tautomeric Preferences of Amine-Adenine Tautomers

Anna Jezuita,[a,]* Paweł Andrzej Wieczorkiewicz,[b] Halina Szatylowicz,[b,]* Tadeusz Marek Krygowski[c]

[a] *Faculty of Science and Technology, Jan Dlugosz University in Czestochowa, Al. Armii Krajowej 13/15, 42-200 Czestochowa, Poland. Email: a.jezuita@ujd.edu.pl*
[b] *Faculty of Chemistry, Warsaw University of Technology, Noakowskiego 3, 00-664 Warsaw, Poland. Email: halina@ch.pw.edu.pl*
[c] *Department of Chemistry, Warsaw University, Pasteura 1, 02-093 Warsaw, Poland.*

Supporting Information

**Table S1**	cSAR(X) values for C8-X and C2-X substitution	SI 2
**Table S2**	cSAR(NH_2) values for C8-X and C2-X substitution	SI 4
**Table S3**	The slopes of linear equations and determination coefficients for dependences of cSAR(X) and cSAR(NH_2) on ε	SI 6
**Table S4**	Ranges of cSAR(X) variability for ε <10 and ε > 10 series in C8-X and C2-X adenine tautomers.	SI 7
**Table S5**	Ranges of cSAR(NH_2) variability for ε <10 and ε > 10 series in C8-X and C2-X adenine tautomers	SI 7
**Table S6**	The slopes of linear equations and determination coefficients for dependences of cSAR(X) and cSAR(NH_2) on 1/ε	SI 8
**Table S7**	C-N Bond lengths	SI 9
**Figure S1**	Dependences of cSAR(X) on 1/ε for C8-X and C2-X substituted 7H tautomers	SI 10
**Figure S2**	Dependences of cSAR(X) on 1/ε for C8-X and C2-X substituted 3H tautomers	SI 11
**Figure S3**	Dependences of cSAR(X) on 1/ε for C8-X and C2-X substituted 1H tautomers	SI 12
**Figure S4**	Dependences of cSAR(NH_2) on 1/ε for C8-X and C2-X substituted 7H tautomers	SI 13
**Figure S5**	Dependences of cSAR(NH_2) on 1/ε for C8-X and C2-X substituted 3H tautomers	SI 14
**Figure S6**	Dependences of cSAR(NH_2) on 1/ε for C8-X and C2-X substituted 1H tautomers	SI 15
**Figure S7**	Dependences of molecular dipole moment on reciprocal of solvent permittivity, 1/ε	SI 16
**Figure S8**	Graphical representations of molecular dipole moment	SI 17
**Figure S9**	Solvation energies, E_{solv}, of unsubstituted and substituted adenine tautomers in studied solvents.	SI 22

Table S1. cSAR(X) values for C8-X (a) and C2-X (b) substitution in different solvents.

(a)		cSAR(X)										
		GP	Tol	ClF	o-Cr	THF	Py	Et-OH	DMSO	H_2O	FA	*range*
	X	**C8-X**										
9H	NO_2	-0.037	-0.062	-0.076	-0.081	-0.082	-0.087	-0.090	-0.092	-0.093	-0.093	**0.056**
	H	0.139	0.150	0.156	0.159	0.159	0.162	0.163	0.164	0.164	0.165	**0.025**
	NH_2	0.233	0.259	0.274	0.280	0.281	0.287	0.291	0.293	0.294	0.294	**0.061**
	range	***0.270***	***0.321***	***0.350***	***0.361***	***0.363***	***0.374***	***0.381***	***0.385***	***0.387***	***0.387***	***0.036***
	av.	*0.112*	*0.116*	*0.118*	*0.119*	*0.120*	*0.121*	*0.121*	*0.122*	*0.122*	*0.122*	***0.048***
	SD	*0.137*	*0.163*	*0.178*	*0.184*	*0.185*	*0.190*	*0.194*	*0.196*	*0.197*	*0.197*	
7H	NO_2	-0.005	-0.027	-0.040	-0.045	-0.046	-0.050	-0.054	-0.055	-0.056	-0.056	**0.052**
	H	0.151	0.161	0.167	0.169	0.170	0.172	0.173	0.174	0.174	0.174	**0.023**
	NH_2	0.253	0.279	0.295	0.300	0.302	0.307	0.311	0.313	0.314	0.314	**0.062**
	range	***0.258***	***0.306***	***0.335***	***0.345***	***0.348***	***0.357***	***0.365***	***0.368***	***0.370***	***0.370***	***0.039***
	av.	*0.133*	*0.138*	*0.141*	*0.142*	*0.142*	*0.143*	*0.143*	*0.144*	*0.144*	*0.144*	***0.046***
	SD	*0.130*	*0.154*	*0.169*	*0.174*	*0.175*	*0.181*	*0.184*	*0.186*	*0.187*	*0.187*	
3H	NO_2	-0.066	-0.119	-0.148	-0.159	-0.161	-0.172	-0.180	-0.184	-0.186	-0.187	**0.120**
	H	0.112	0.100	0.093	0.090	0.090	0.087	0.086	0.085	0.084	0.084	**0.028**
	NH_2	0.238	0.219	0.216	0.215	0.214	0.213	0.212	0.211	0.211	0.211	**0.027**
	range	***0.304***	***0.338***	***0.364***	***0.374***	***0.375***	***0.385***	***0.392***	***0.395***	***0.397***	***0.398***	***0.093***
	av.	*0.095*	*0.067*	*0.054*	*0.049*	*0.048*	*0.043*	*0.039*	*0.037*	*0.036*	*0.036*	***0.059***
	SD	*0.153*	*0.171*	*0.185*	*0.190*	*0.191*	*0.196*	*0.200*	*0.202*	*0.203*	*0.203*	
1H	NO_2	-0.051	-0.113	-0.144	-0.156	-0.159	-0.171	-0.179	-0.184	-0.186	-0.187	**0.135**
	H	0.121	0.104	0.093	0.090	0.089	0.085	0.082	0.081	0.080	0.080	**0.041**
	NH_2	0.237	0.226	0.217	0.213	0.212	0.209	0.206	0.205	0.204	0.204	**0.033**
	range	***0.288***	***0.339***	***0.361***	***0.369***	***0.371***	***0.380***	***0.385***	***0.389***	***0.390***	***0.391***	***0.102***
	av.	*0.102*	*0.072*	*0.055*	*0.049*	*0.048*	*0.041*	*0.036*	*0.034*	*0.033*	*0.032*	***0.070***
	SD	*0.145*	*0.172*	*0.184*	*0.188*	*0.189*	*0.193*	*0.196*	*0.198*	*0.199*	*0.199*	

(b)		cSAR(X)										
		GP	Tol	ClF	o-Cr	THF	Py	Et-OH	DMSO	H_2O	FA	*range*
	X	**C2-X**										
9H	NO_2	-0.030	-0.060	-0.084	-0.090	-0.092	-0.099	-0.103	-0.106	-0.107	-0.108	**0.077**
	H	0.133	0.126	0.123	0.121	0.121	0.120	0.119	0.119	0.118	0.118	**0.014**
	NH_2	0.230	0.231	0.231	0.231	0.231	0.231	0.231	0.230	0.230	0.230	**0.001**
	range	***0.260***	***0.291***	***0.315***	***0.321***	***0.323***	***0.330***	***0.334***	***0.336***	***0.337***	***0.338***	***0.076***
	av.	*0.111*	*0.099*	*0.090*	*0.087*	*0.087*	*0.084*	*0.082*	*0.081*	*0.081*	*0.080*	***0.031***
	SD	*0.132*	*0.147*	*0.160*	*0.163*	*0.164*	*0.168*	*0.170*	*0.171*	*0.172*	*0.172*	
7H	NO_2	-0.008	-0.056	-0.079	-0.087	-0.089	-0.100	-0.106	-0.109	-0.110	-0.111	**0.103**
	H	0.134	0.126	0.121	0.118	0.118	0.116	0.114	0.113	0.113	0.113	**0.021**
	NH_2	0.229	0.226	0.223	0.221	0.221	0.219	0.217	0.217	0.216	0.216	**0.013**
	range	***0.237***	***0.282***	***0.302***	***0.308***	***0.310***	***0.319***	***0.323***	***0.326***	***0.326***	***0.327***	***0.090***
	av.	*0.118*	*0.099*	*0.088*	*0.084*	*0.083*	*0.078*	*0.075*	*0.074*	*0.073*	*0.073*	***0.046***
	SD	*0.119*	*0.143*	*0.153*	*0.157*	*0.158*	*0.163*	*0.165*	*0.166*	*0.167*	*0.167*	
3H	NO_2	0.014	0.005	0.001	0.000	-0.001	-0.002	-0.002	-0.003	-0.003	-0.003	**0.017**
	H	0.165	0.181	0.190	0.194	0.194	0.198	0.200	0.201	0.202	0.202	**0.037**
	NH_2	0.265	0.301	0.320	0.327	0.329	0.336	0.340	0.343	0.344	0.345	**0.080**
	range	***0.251***	***0.296***	***0.319***	***0.327***	***0.330***	***0.338***	***0.342***	***0.346***	***0.347***	***0.348***	***0.063***
	av.	*0.148*	*0.162*	*0.170*	*0.173*	*0.174*	*0.177*	*0.179*	*0.180*	*0.181*	*0.181*	***0.045***
	SD	*0.126*	*0.149*	*0.161*	*0.165*	*0.166*	*0.170*	*0.172*	*0.174*	*0.175*	*0.175*	
1H	NO_2	-0.010	-0.029	-0.037	-0.040	-0.040	-0.043	-0.045	-0.045	-0.045	-0.046	**0.035**
	H	0.150	0.165	0.174	0.177	0.178	0.181	0.183	0.185	0.185	0.185	**0.035**
	NH_2	0.236	0.267	0.284	0.292	0.294	0.302	0.307	0.310	0.311	0.312	**0.076**
	range	***0.246***	***0.296***	***0.321***	***0.332***	***0.334***	***0.345***	***0.352***	***0.355***	***0.356***	***0.358***	***0.041***
	av.	*0.125*	*0.134*	*0.140*	*0.143*	*0.144*	*0.147*	*0.148*	*0.150*	*0.150*	*0.150*	***0.049***
	SD	*0.125*	*0.150*	*0.163*	*0.169*	*0.170*	*0.175*	*0.179*	*0.180*	*0.181*	*0.181*	

Table S2. cSAR(NH_2) values for C8-X (a) and C2-X (b) substitution in different solvents.

(a)		cSAR(NH_2)										
		GP	Tol	ClF	o-Cr	THF	Py	Et-OH	DMSO	H_2O	FA	*range*
	X	**C8-X**										
9H	NO_2	0.289	0.314	0.326	0.331	0.332	0.336	0.339	0.340	0.341	0.341	**0.053**
	H	0.241	0.254	0.259	0.261	0.261	0.263	0.264	0.264	0.264	0.264	**0.024**
	NH_2	0.209	0.215	0.216	0.216	0.216	0.216	0.216	0.215	0.215	0.215	**0.007**
	range	***0.080***	***0.099***	***0.110***	***0.115***	***0.116***	***0.120***	***0.123***	***0.125***	***0.126***	***0.126***	***0.046***
	av.	*0.246*	*0.261*	*0.267*	*0.269*	*0.270*	*0.272*	*0.273*	*0.273*	*0.273*	*0.273*	***0.028***
	SD	*0.033*	*0.041*	*0.045*	*0.047*	*0.048*	*0.049*	*0.051*	*0.051*	*0.052*	*0.052*	
7H	NO_2	0.229	0.273	0.302	0.311	0.314	0.322	0.328	0.332	0.333	0.334	**0.105**
	H	0.184	0.215	0.234	0.242	0.244	0.253	0.259	0.262	0.264	0.264	**0.080**
	NH_2	0.160	0.180	0.194	0.200	0.201	0.207	0.212	0.214	0.215	0.216	**0.056**
	range	***0.069***	***0.093***	***0.108***	***0.111***	***0.113***	***0.115***	***0.116***	***0.118***	***0.118***	***0.118***	***0.049***
	av.	*0.191*	*0.222*	*0.243*	*0.251*	*0.253*	*0.261*	*0.266*	*0.269*	*0.271*	*0.271*	***0.080***
	SD	*0.035*	*0.047*	*0.054*	*0.056*	*0.057*	*0.058*	*0.059*	*0.059*	*0.059*	*0.059*	
3H	NO_2	0.316	0.345	0.359	0.365	0.366	0.371	0.375	0.377	0.378	0.378	**0.062**
	H	0.270	0.287	0.296	0.299	0.299	0.302	0.304	0.305	0.305	0.306	**0.036**
	NH_2	0.222	0.229	0.234	0.236	0.236	0.237	0.238	0.239	0.239	0.239	**0.017**
	range	***0.094***	***0.116***	***0.125***	***0.129***	***0.130***	***0.134***	***0.137***	***0.138***	***0.139***	***0.139***	***0.045***
	av.	*0.269*	*0.287*	*0.296*	*0.300*	*0.300*	*0.304*	*0.306*	*0.307*	*0.307*	*0.308*	***0.038***
	SD	*0.047*	*0.058*	*0.063*	*0.065*	*0.065*	*0.067*	*0.068*	*0.069*	*0.069*	*0.070*	
1H	NO_2	0.301	0.366	0.395	0.405	0.408	0.418	0.426	0.430	0.432	0.433	**0.132**
	H	0.244	0.294	0.322	0.333	0.335	0.346	0.354	0.358	0.360	0.361	**0.117**
	NH_2	0.186	0.227	0.254	0.265	0.267	0.277	0.284	0.288	0.290	0.290	**0.104**
	range	***0.115***	***0.139***	***0.141***	***0.140***	***0.141***	***0.141***	***0.142***	***0.142***	***0.142***	***0.143***	***0.028***
	av.	*0.244*	*0.296*	*0.324*	*0.334*	*0.336*	*0.347*	*0.354*	*0.359*	*0.361*	*0.361*	***0.118***
	SD	*0.057*	*0.069*	*0.070*	*0.070*	*0.070*	*0.071*	*0.071*	*0.071*	*0.071*	*0.071*	

(b)		cSAR(NH_2)										
		GP	Tol	ClF	o-Cr	THF	Py	Et-OH	DMSO	H_2O	FA	*range*
	X	**C2-X**										
9H	NO_2	0.285	0.305	0.314	0.317	0.318	0.321	0.323	0.324	0.325	0.325	**0.040**
	H	0.241	0.254	0.259	0.261	0.261	0.263	0.264	0.264	0.264	0.264	**0.024**
	NH_2	0.226	0.235	0.239	0.240	0.240	0.241	0.242	0.242	0.242	0.242	**0.016**
	range	***0.059***	***0.070***	***0.075***	***0.077***	***0.078***	***0.080***	***0.081***	***0.082***	***0.083***	***0.083***	***0.024***
	av.	*0.251*	*0.264*	*0.271*	*0.273*	*0.273*	*0.275*	*0.276*	*0.277*	*0.277*	*0.277*	***0.027***
	SD	*0.030*	*0.036*	*0.039*	*0.040*	*0.040*	*0.041*	*0.042*	*0.042*	*0.043*	*0.043*	
7H	NO_2	0.231	0.271	0.296	0.305	0.308	0.318	0.327	0.330	0.332	0.332	**0.102**
	H	0.184	0.215	0.234	0.242	0.244	0.253	0.259	0.262	0.264	0.264	**0.080**
	NH_2	0.173	0.201	0.218	0.226	0.227	0.235	0.240	0.243	0.244	0.245	**0.072**
	range	***0.058***	***0.070***	***0.078***	***0.079***	***0.081***	***0.083***	***0.087***	***0.087***	***0.088***	***0.087***	***0.030***
	av.	*0.196*	*0.229*	*0.249*	*0.258*	*0.260*	*0.269*	*0.275*	*0.278*	*0.280*	*0.280*	***0.084***
	SD	*0.031*	*0.037*	*0.041*	*0.042*	*0.042*	*0.044*	*0.046*	*0.046*	*0.046*	*0.046*	
3H	NO_2	0.303	0.329	0.341	0.345	0.346	0.350	0.353	0.355	0.355	0.356	**0.052**
	H	0.270	0.287	0.296	0.299	0.299	0.302	0.304	0.305	0.305	0.306	**0.036**
	NH_2	0.260	0.274	0.280	0.282	0.283	0.285	0.286	0.286	0.287	0.287	**0.027**
	range	***0.043***	***0.055***	***0.061***	***0.063***	***0.063***	***0.065***	***0.067***	***0.069***	***0.068***	***0.069***	***0.025***
	av.	*0.277*	*0.296*	*0.306*	*0.309*	*0.309*	*0.312*	*0.314*	*0.315*	*0.316*	*0.316*	***0.039***
	SD	*0.023*	*0.029*	*0.032*	*0.033*	*0.033*	*0.034*	*0.035*	*0.035*	*0.035*	*0.035*	
1H	NO_2	0.310	0.363	0.389	0.398	0.400	0.410	0.420	0.420	0.422	0.422	**0.112**
	H	0.244	0.294	0.322	0.333	0.335	0.346	0.354	0.358	0.360	0.361	**0.117**
	NH_2	0.228	0.273	0.302	0.312	0.314	0.325	0.332	0.336	0.338	0.339	**0.111**
	range	***0.082***	***0.090***	***0.087***	***0.086***	***0.086***	***0.085***	***0.088***	***0.084***	***0.084***	***0.083***	***0.006***
	av.	*0.261*	*0.310*	*0.338*	*0.348*	*0.350*	*0.360*	*0.368*	*0.371*	*0.373*	*0.374*	***0.113***
	SD	*0.043*	*0.047*	*0.045*	*0.045*	*0.045*	*0.044*	*0.046*	*0.043*	*0.043*	*0.043*	

Table S3. The slopes of linear equations, *a*, and determination coefficients, R^2, for dependences of cSAR(X) (a) and cSAR(NH_2) (b) on ε for tautomers of adenine.

a)		C8-X				C2-X			
		$a\cdot10^4$	R^2	$a\cdot10^4$	R^2	$a\cdot10^4$	R^2	$a\cdot10^4$	R^2
		ε <10		ε > 10		ε <10		ε > 10	
		cSAR(X) on ε							
9H	NO_2	-63	0.855	-0.6	0.738	-90	0.895	-0.8	0.745
	H	28	0.870	0.3	0.734	-16	0.855	-0.1	0.734
	NH_2	69	0.869	0.6	0.735	0.6	0.193	-0.04	0.821
7H	NO_2	-59	0.873	-0.5	0.741	-113	0.838	-1.0	0.719
	H	26	0.854	0.2	0.744	-23	0.895	-0.3	0.733
	NH_2	70	0.874	0.6	0.750	-11	0.960	-0.3	0.751
3H	NO_2	-134	0.863	-1.0	0.739	-20	0.794	-0.1	0.722
	H	-32	0.862	-0.3	0.737	41	0.863	0.4	0.737
	NH_2	-30	0.672	-0.2	0.739	89	0.857	0.8	0.738
1H	NO_2	-149	0.848	-1.0	0.740	-41	0.810	-0.2	0.528
	H	-46	0.870	-0.5	0.748	39	0.875	0.4	0.732
	NH_2	-36	0.927	-0.5	0.743	82	0.878	0.9	0.740

b)		C8-X				C2-X			
		$a\cdot10^4$	R^2	$a\cdot10^4$	R^2	$a\cdot10^4$	R^2	$a\cdot10^4$	R^2
		ε < 10		ε > 10		ε < 10			
		cSAR(NH_2) on ε							
9H	NO_2	60	0.843	0.50	0.734	53	0.897	0.30	0.725
	H	29	0.805	0.10	0.710	34	0.886	0.10	0.710
	NH_2	8	0.621	0.06	0.802	22	0.891	0.09	0.488
7H	NO_2	121	0.883	1.0	0.742	110	0.880	1.0	0.662
	H	86	0.895	1.0	0.727	86	0.895	1.0	0.727
	NH_2	59	0.906	0.8	0.727	77	0.889	0.9	0.743
3H	NO_2	70	0.848	0.6	0.737	60	0.836	0.6	0.734
	H	41	0.837	0.3	0.732	41	0.837	0.3	0.732
	NH_2	20	0.890	0.1	0.717	32	0.822	0.2	0.724
1H	NO_2	146	0.899	1.0	0.739	125	0.842	0.9	0.540
	H	129	0.864	1.0	0.755	129	0.864	1.0	0.755
	NH_2	116	0.887	1.0	0.738	123	0.882	1.0	0.753

Table S4. Ranges of cSAR(X) variability and their ratios ($\varepsilon_I/\varepsilon_{II}$) for media with $\varepsilon < 10$ (ε_I) and $\varepsilon > 10$ (ε_{II}) for C8-X and C2-X substituted adenine tautomers.

		cSAR(X)					
		C8-X			C2-X		
		ε_I	ε_{II}	$\varepsilon_I/\varepsilon_{II}$	ε_I	ε_{II}	$\varepsilon_I/\varepsilon_{II}$
9H	NO_2	0.045	0.006	6.98	0.062	0.009	6.91
	H	0.020	0.003	6.31	0.011	0.002	7.40
	NH_2	0.049	0.007	6.88	0.001	0.000	2.70
7H	NO_2	0.041	0.006	6.83	0.082	0.011	7.56
	H	0.019	0.003	6.93	0.016	0.003	5.43
	NH_2	0.049	0.007	7.13	0.008	0.003	3.01
3H	NO_2	0.095	0.015	6.51	0.015	0.001	12.50
	H	0.022	0.003	6.82	0.029	0.004	6.75
	NH_2	0.024	0.002	12.70	0.064	0.009	6.98
1H	NO_2	0.107	0.016	6.68	0.030	0.003	10.64
	H	0.032	0.005	6.08	0.028	0.004	6.41
	NH_2	0.025	0.005	4.84	0.058	0.010	5.98

Table S5. Ranges of cSAR(NH_2) variability, Δ, and their percentages for $\varepsilon < 10$ and $\varepsilon > 10$ series in C8-X and C2-X adenine tautomers.

		cSAR(NH_2)							
		C8-X				C2-X			
		$\varepsilon < 10$		$\varepsilon > 10$		$\varepsilon < 10$		$\varepsilon > 10$	
		Δ	%	Δ	%	Δ	%	Δ	%
9H	NO_2	0.043	88.7	0.005	11.3	0.033	89.9	0.004	10.1
	H	0.021	93.2	0.002	6.8	0.021	93.2	0.002	6.8
	NH_2	0.007	92.7	0.001	7.3	0.014	91.7	0.001	8.3
7H	NO_2	0.084	87.9	0.012	12.1	0.077	84.6	0.014	15.4
	H	0.060	84.5	0.011	15.5	0.060	84.5	0.011	15.5
	NH_2	0.041	82.7	0.009	17.3	0.054	84.5	0.010	15.5
3H	NO_2	0.050	88.1	0.007	11.9	0.043	89.4	0.005	10.6
	H	0.030	89.4	0.004	10.6	0.030	89.4	0.004	10.6
	NH_2	0.014	89.5	0.002	10.5	0.023	90.9	0.002	9.1
1H	NO_2	0.107	88.2	0.014	11.8	0.090	87.9	0.012	12.1
	H	0.091	85.6	0.015	14.4	0.091	85.6	0.015	14.4
	NH_2	0.081	85.8	0.013	14.2	0.086	85.6	0.014	14.4

Table S6. The slopes of linear equations, *a*, and determination coefficients, R^2, for dependences of cSAR(X) and cSAR(NH_2) on 1/ε for C8-X and C2-X substituted adenine tautomers. a_X/a_{NH2} denote the ratio between cSAR(X) vs 1/ε and cSAR(NH_2) vs 1/ε slopes.

	X	C8-X					C2-X				
		a	R^2	*a*	R^2	a_X/a_{NH2}	*a*	R^2	*a*	R^2	a_X/a_{NH2}
		cSAR(X) vs 1/ ε		cSAR(NH_2) vs 1/ε			cSAR(X) vs 1/ ε		cSAR(NH_2) vs 1/ε		
9H	NO_2	0.057	0.976	-0.054	0.984	-0.94	0.080	0.961	-0.041	0.991	-0.51
	H	-0.026	0.966	-0.024	0.999	0.94	0.015	0.979	-0.024	0.999	-1.68
	NH_2	-0.063	0.971	-0.006	0.827	0.09	0.000	0.006	-0.017	0.995	-
7H	NO_2	0.053	0.970	-0.108	0.970	-2.03	0.105	0.977	-0.104	0.950	-0.99
	H	-0.024	0.978	-0.082	0.941	3.43	0.022	0.943	-0.082	0.941	-3.74
	NH_2	-0.063	0.971	-0.057	0.930	0.90	0.013	0.847	-0.073	0.947	-5.57
3H	NO_2	0.123	0.970	-0.063	0.981	-0.52	0.018	0.999	-0.053	0.988	-3.01
	H	0.029	0.973	-0.037	0.988	-1.27	-0.038	0.972	-0.037	0.988	0.97
	NH_2	0.027	0.986	-0.018	0.970	-0.66	-0.081	0.976	-0.028	0.994	0.34
1H	NO_2	0.137	0.976	-0.133	0.987	-0.97	0.036	0.996	-0.114	0.980	-3.18
	H	0.042	0.965	-0.119	0.967	-2.82	-0.036	0.964	-0.119	0.967	3.31
	NH_2	0.034	0.921	-0.107	0.957	-3.10	-0.077	0.956	-0.113	0.960	1.47

Table S7. C-N Bond lengths (in Å) in the gas phase (GP) and formamide (FA), their difference, Δ, and type of proximity (see Scheme 2)

X=NH_2		GP		FA		ΔC^X-N	ΔC^6-N	proximity type
		C^X-NH_2	C^6-N	C^X-NH_2	C^6-N			
9H	**C2**	1.3801	1.3653	1.3812	1.3628	*0.0011*	*-0.0025*	I
7H		1.3813	1.3887	1.3848	1.3699	*0.0035*	*-0.0188*	I
3H		1.3868	1.3554	1.3668	1.3512	*-0.0199*	*-0.0042*	II
1H		1.3975	1.3790	1.3770	1.3515	*-0.0204*	*-0.0274*	II
9H	**C8**	1.3860	1.3707	1.3718	1.3703	*-0.0141*	*-0.0003*	II
7H		1.3803	1.3916	1.3655	1.3771	*-0.0148*	*-0.0145*	II
3H		1.3744	1.3680	1.3775	1.3645	*0.0031*	*-0.0035*	I
1H		1.3695	1.3905	1.3780	1.3653	*0.0085*	*-0.0252*	I
X=NO_2		GP		FA		ΔC^X-N	ΔC^6-N	proximity type
		C^X-NO_2	C^6-N	C^X-NO_2	C^6-N			
9H	**C2**	1.5311	1.3540	1.5213	1.3475	*-0.0099*	*-0.0065*	I
7H		1.5125	1.3761	1.5246	1.3508	*0.0122*	*-0.0253*	I
3H		1.4941	1.3506	1.4905	1.3431	*-0.0037*	*-0.0075*	II
1H		1.4799	1.3566	1.4725	1.3367	*-0.0074*	*-0.0198*	II
9H	**C8**	1.4512	1.3530	1.4393	1.3451	*-0.0118*	*-0.0079*	II
7H		1.4613	1.3749	1.4492	1.3506	*-0.0121*	*-0.0243*	II
3H		1.4755	1.3477	1.4549	1.3391	*-0.0206*	*-0.0086*	I
1H		1.4804	1.3605	1.4589	1.3357	*-0.0215*	*-0.0248*	I

X=H	GP C^6-N	FA C^6-N	ΔC^6-N
9H	1.3616	1.3703	*0.0087*
7H	1.3861	1.3771	*-0.0090*
3H	1.3542	1.3645	*0.0103*
1H	1.3752	1.3653	*-0.0099*

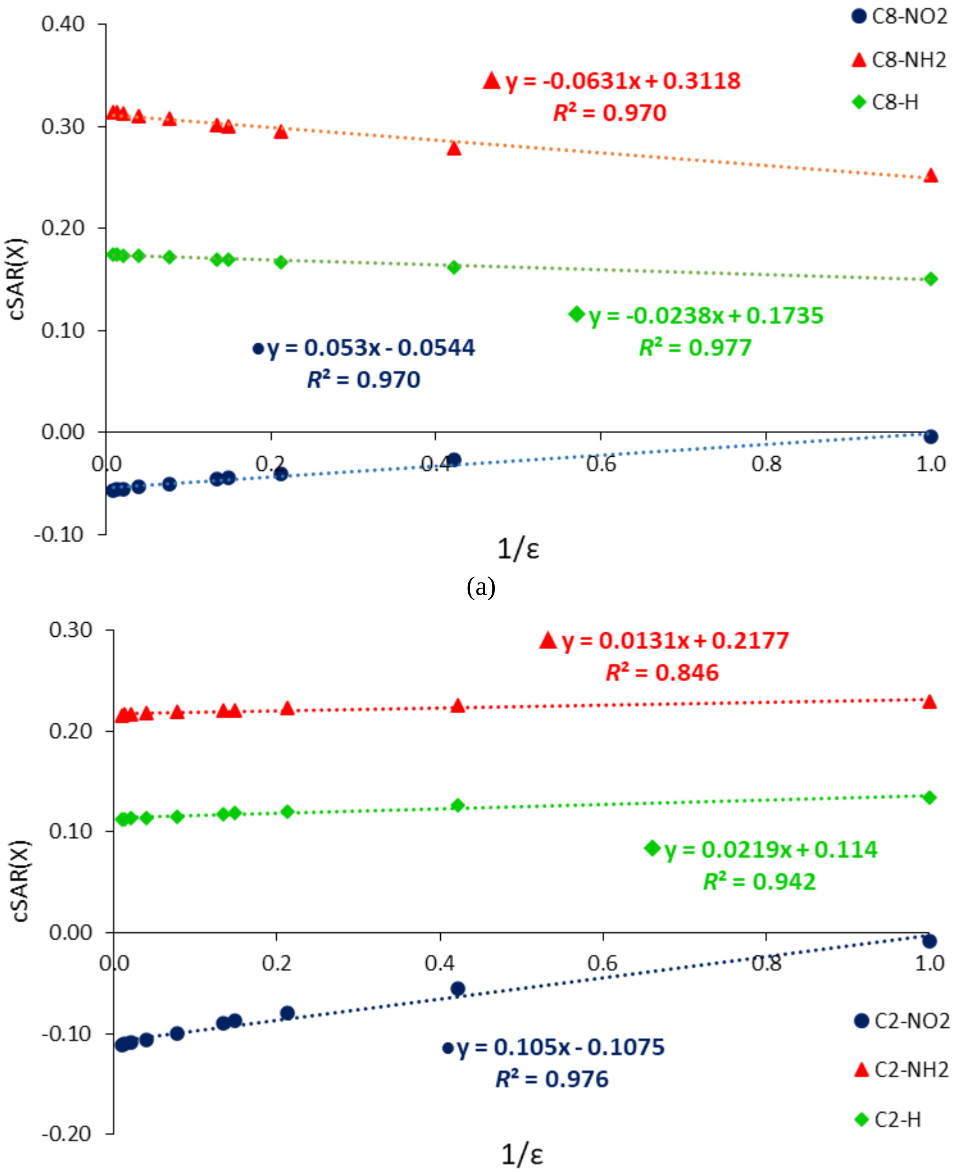

(a)

(b)

Figure S1. Dependences of cSAR(X) on 1/ε for C8-X (a) and C2-X (b) substituted 7H tautomers (X = NH_2, H and NO_2).

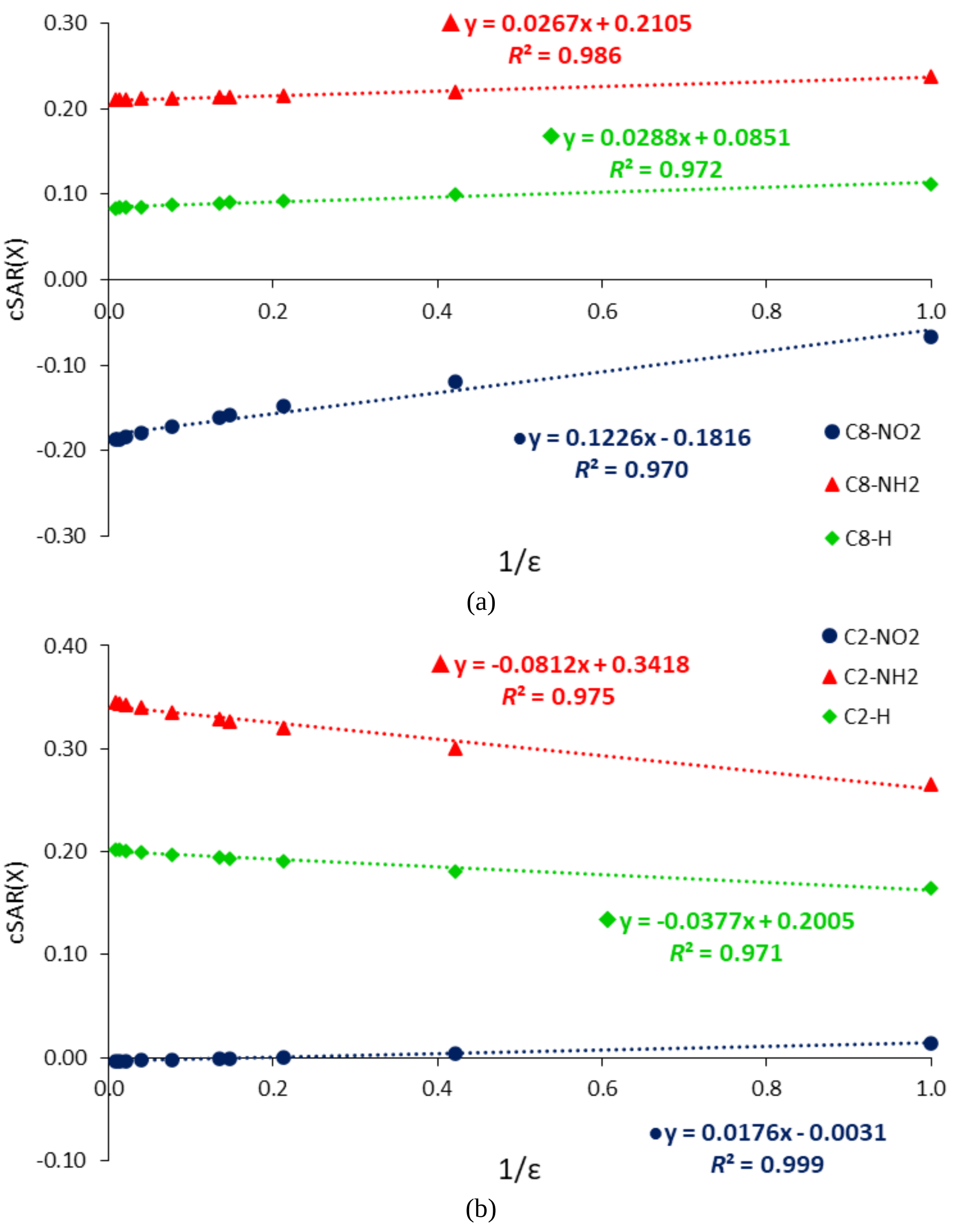

Figure S2. Dependences of cSAR(X) on 1/ε for C8-X (a) and C2-X (b) substituted 3H tautomers (X = NH_2, H and NO_2).

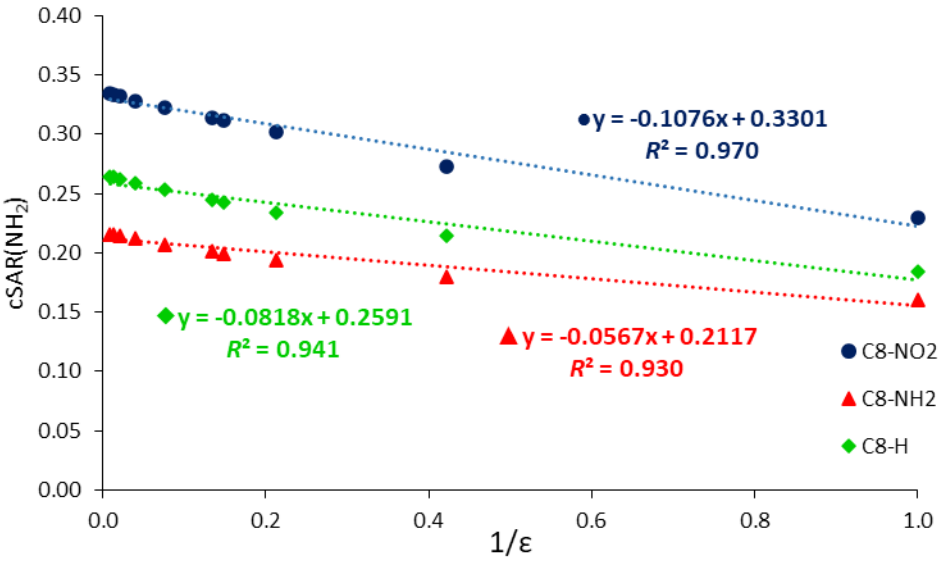

(a)

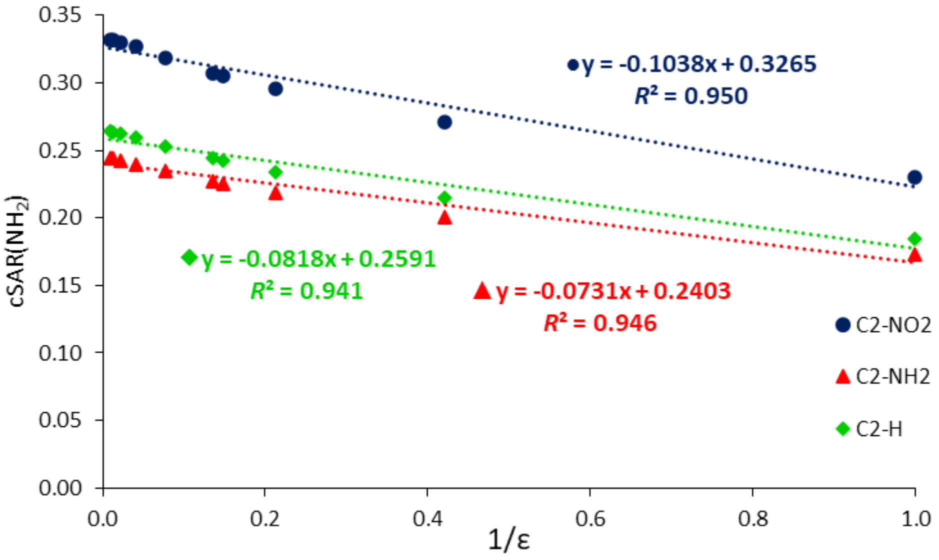

(b)

Figure S3. Dependences of cSAR(X) on 1/ε for C8-X (a) and C2-X (b) substituted 1H tautomers (X = NH_2, H and NO_2).

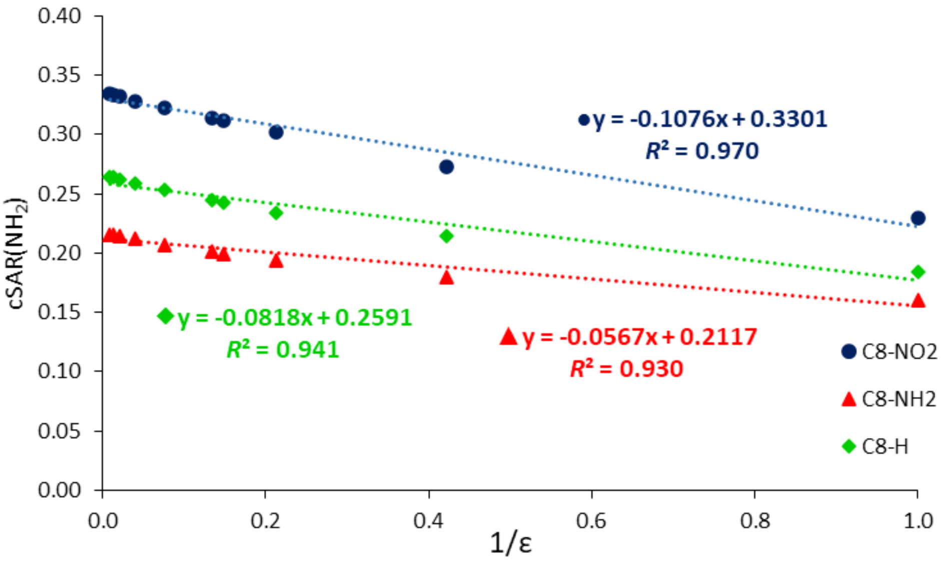

(a)

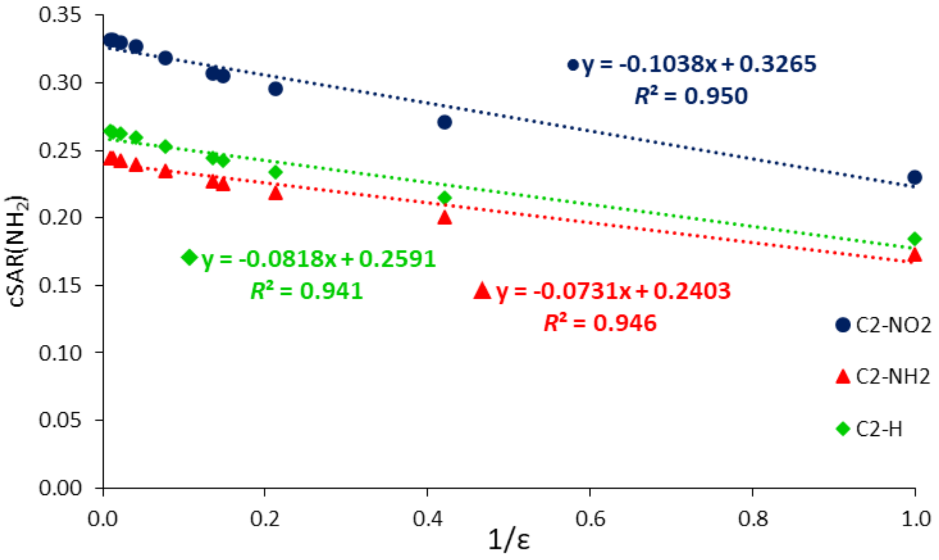

(b)

Figure S4. Dependences of cSAR(NH_2) on 1/ε for C8-X (a) and C2-X (b) substituted 7H tautomers (X = NH_2, H and NO_2).

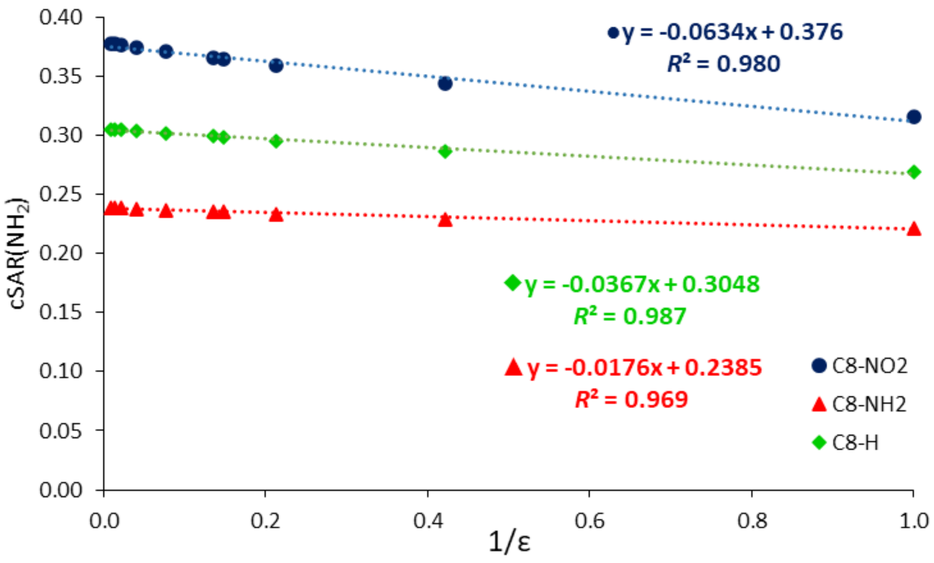

(a)

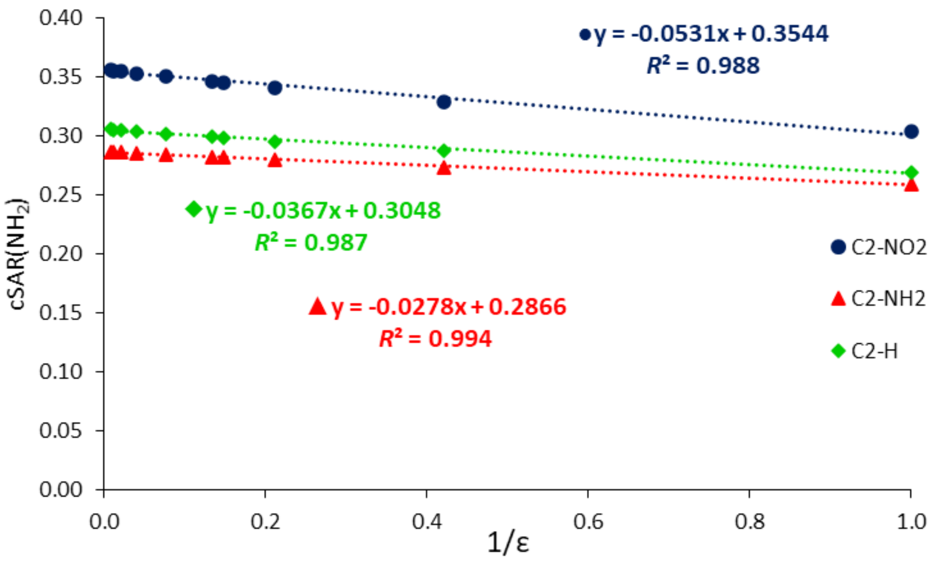

(b)

Figure S5. Dependences of cSAR(NH_2) on 1/ε for C8-X (a) and C2-X (b) substituted 3H tautomers (X = NH_2, H and NO_2).

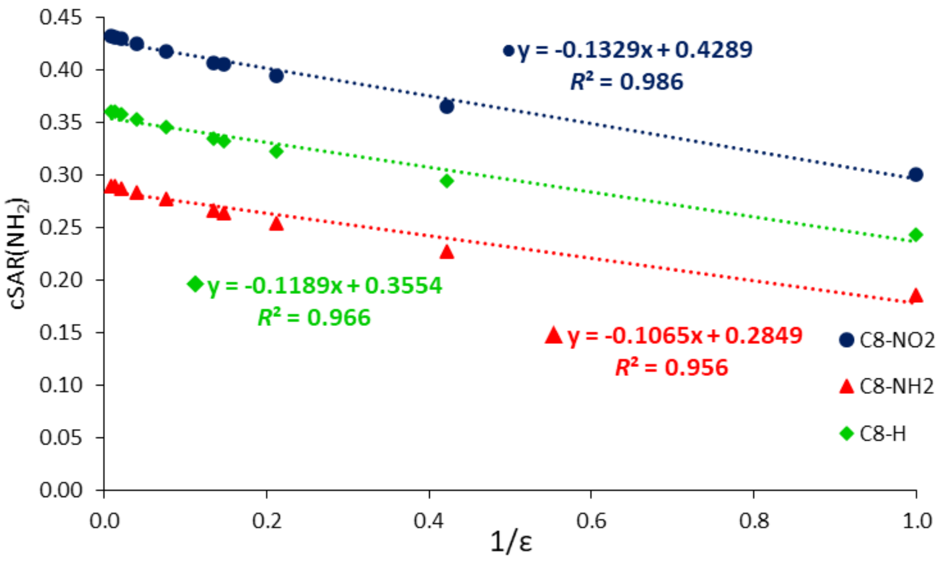

(a)

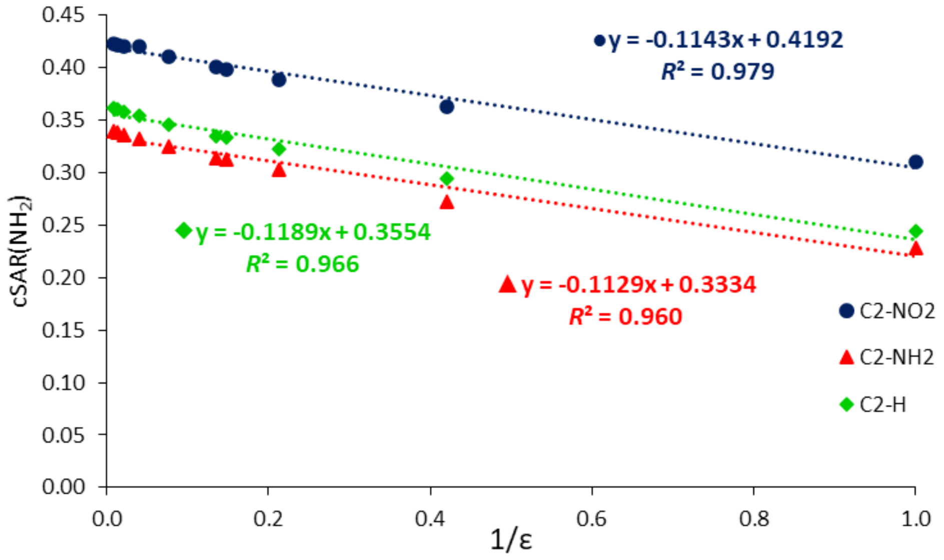

(b)

Figure S6. Dependences of cSAR(NH_2) on 1/ε for C8-X (a) and C2-X (b) substituted 1H tautomers (X = NH_2, H and NO_2).

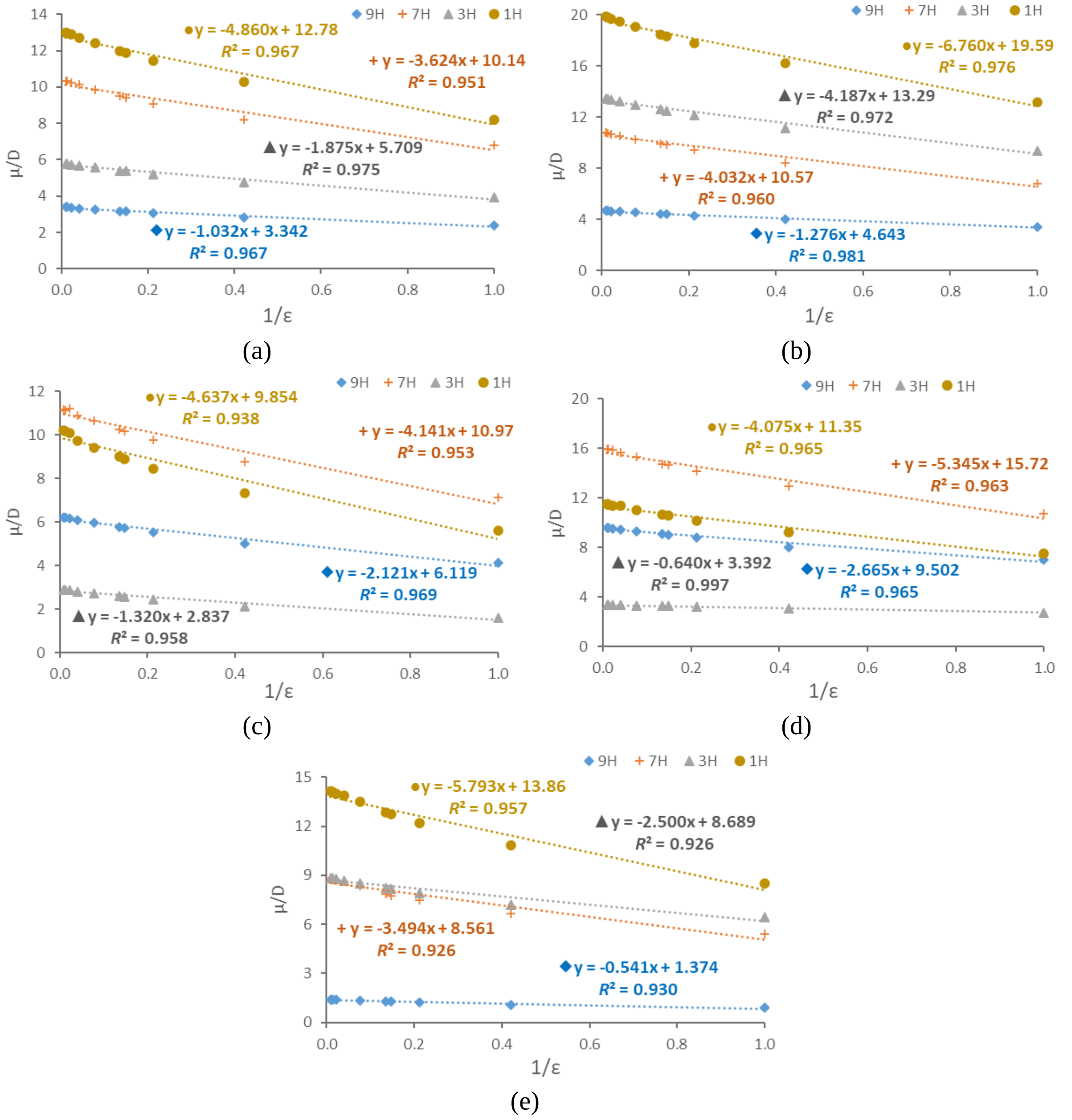

Figure S7. Dependences of molecular dipole moment, μ (in Debye), on reciprocal of solvent permittivity, 1/ε, for unsubstituted (a), C8-NO_2 (b), C8-NH_2 (c), C2-NO_2 (d), and C2-NH_2 (e) substituted adenine amino tautomers.

Figure S8. Graphical representations of molecular dipole moment for unsubstituted, as well as C2 and C8 substituted (X = NH_2 or NO_2) adenine tautomers in two extreme cases of environment dielectric constant: gas phase (ε =1.00) and formamide (ε =108.94).

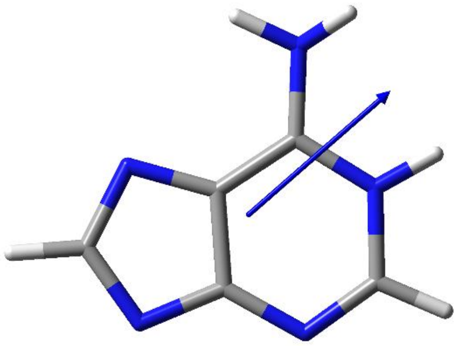

AD 1H gas phase

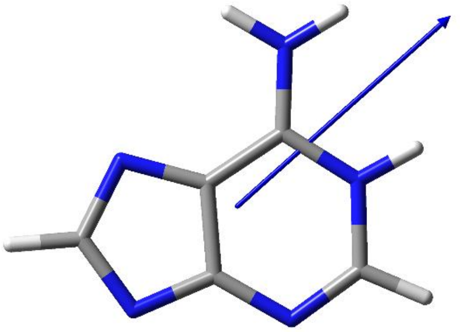

AD 1H formamide

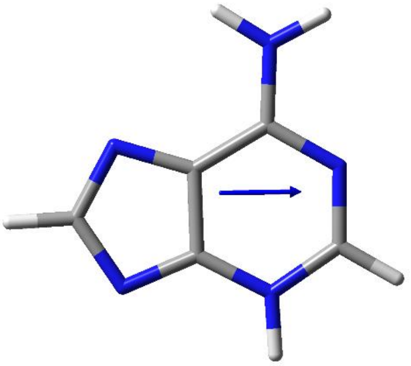

AD 3H gas phase

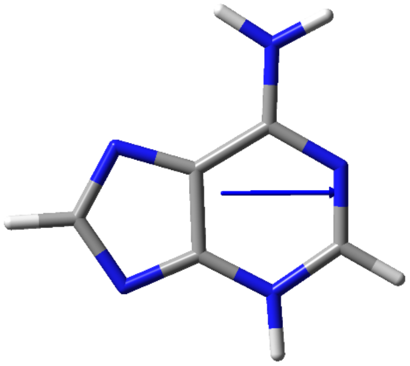

AD 3H formamide

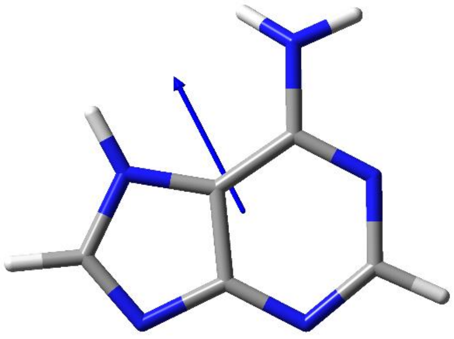

AD 7H gas phase

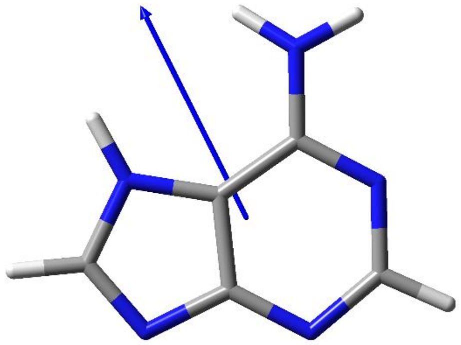

AD 7H formamide

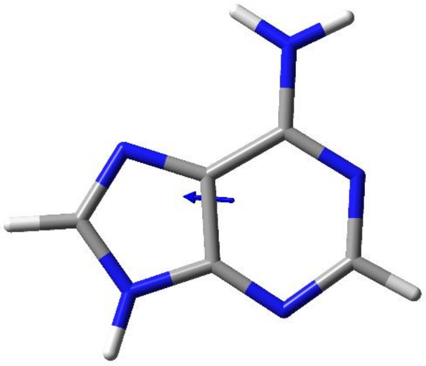

AD 9H gas phase

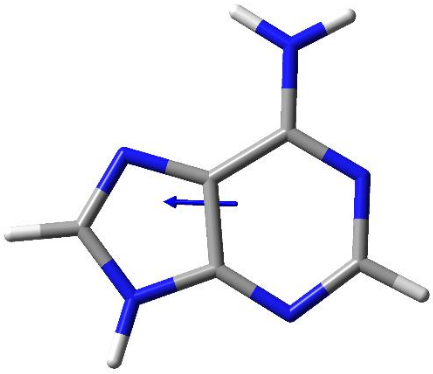

AD 9H formamide

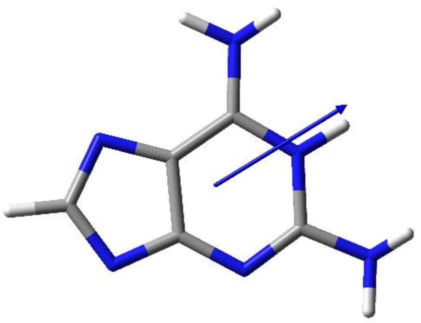

AD 1H C2-NH2 gas phase

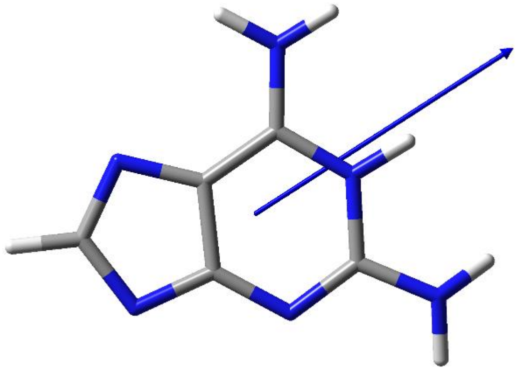

AD 1H C2-NH2 formamide

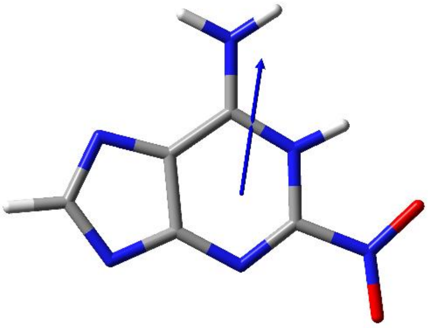

AD 1H C2-NO2 gas phase

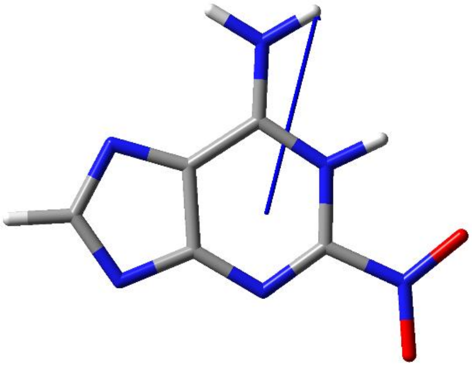

AD 1H C2-NO2 formamide

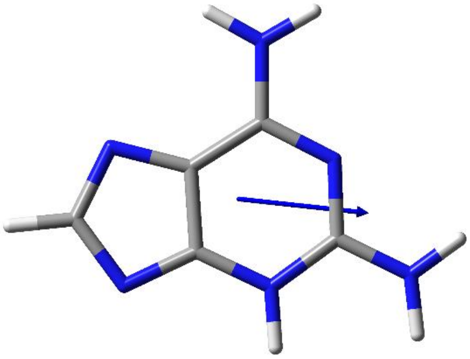

AD 3H C2-NH2 gas phase

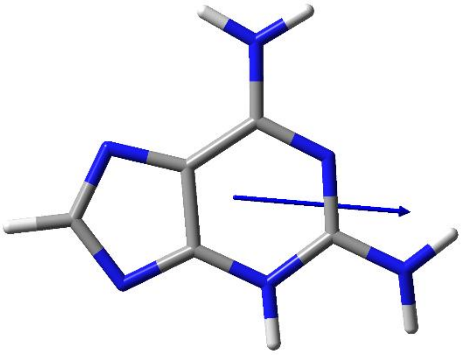

AD 3H C2-NH2 gas phase formamide

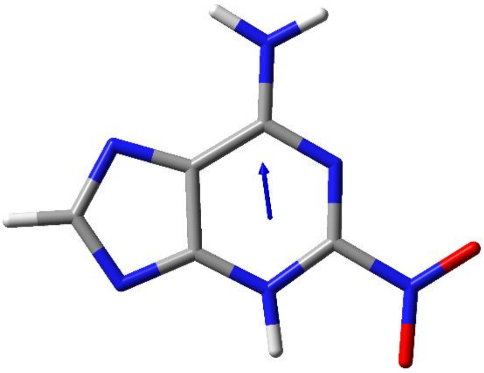

AD 3H C2-NO2 gas phase

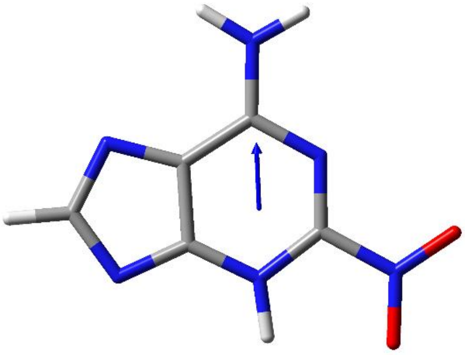

AD 3H C2-NO2 formamide

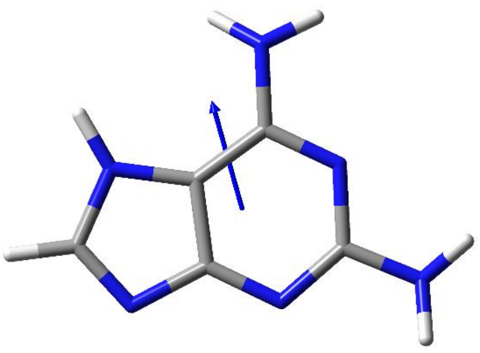

AD 7H C2-NH2 gas phase

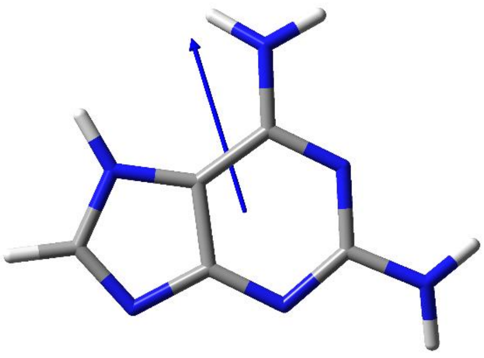

AD 7H C2-NH2 formamide

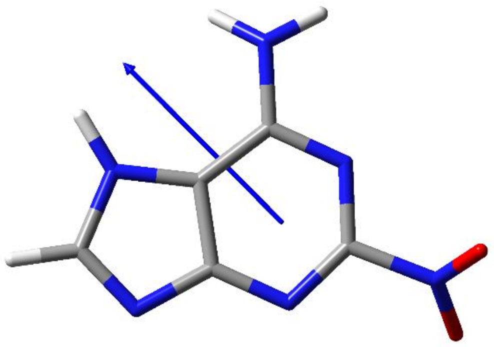

AD 7H C2-NO2 gas phase

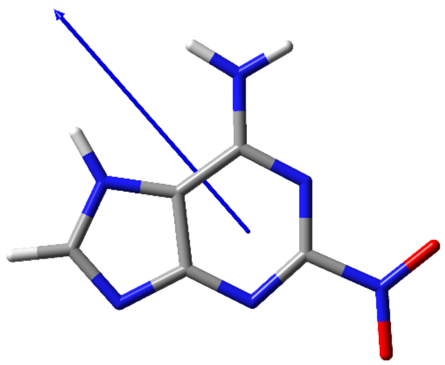

AD 7H C2-NO2 formamide

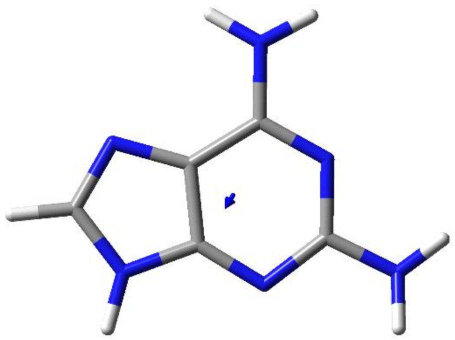

AD 9H C2-NH2 gas phase

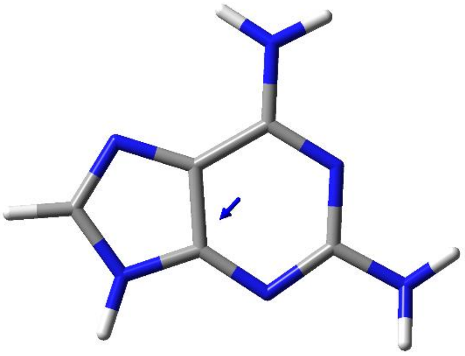

AD 9H C2-NH2 formamide

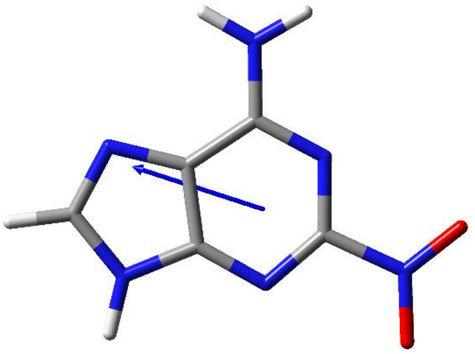

AD 9H C2-NO2 gas phase

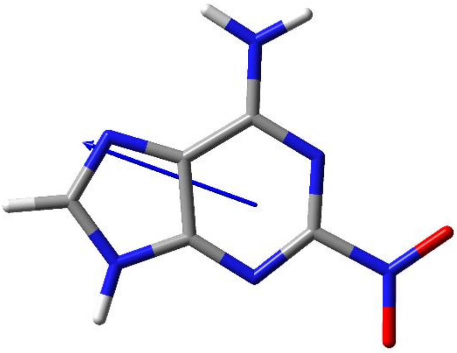

AD 9H C2-NO2 formamide

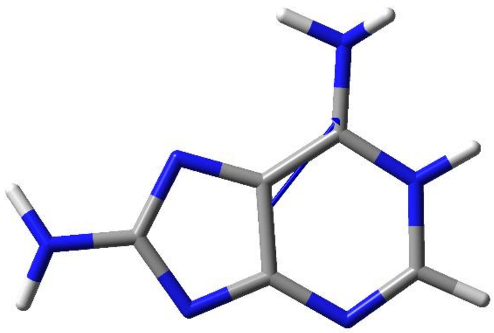

AD 1H C8-NH2 gas phase

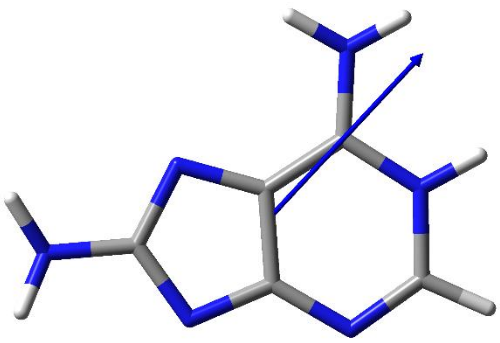

AD 1H C8-NH2 formamide

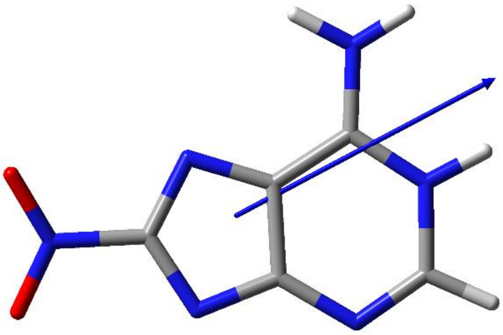

AD 1H C8-NO2 gas phase

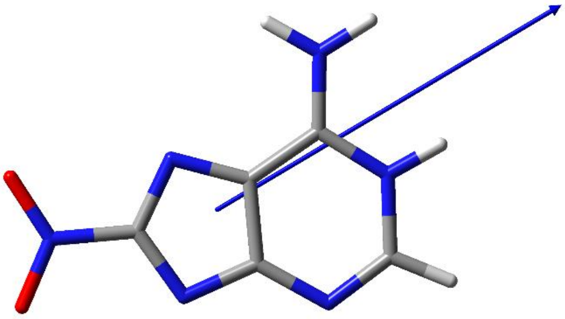

AD 1H C8-NO2 formamide

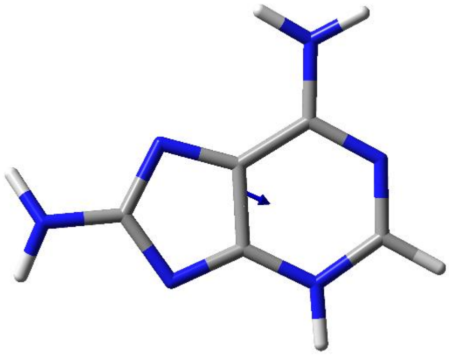

AD 3H C8-NH2 gas phase

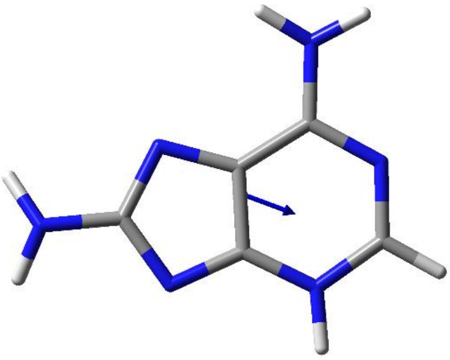

AD 3H C8-NH2 formamide

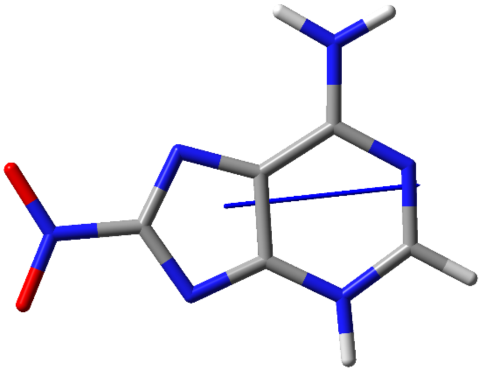

AD 3H C8-NO2 gas phase

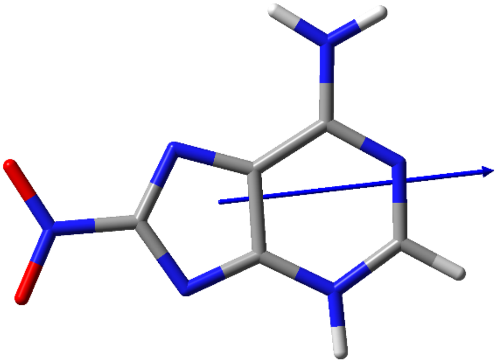

AD 3H C8-NO2 formamide

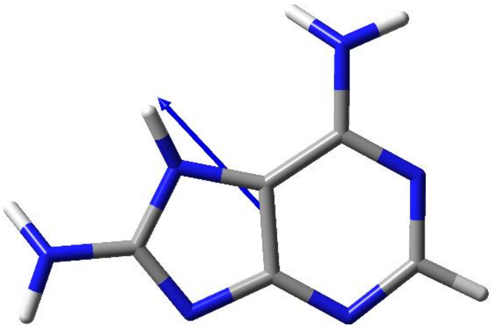

AD 7H C8-NH2 gas phase

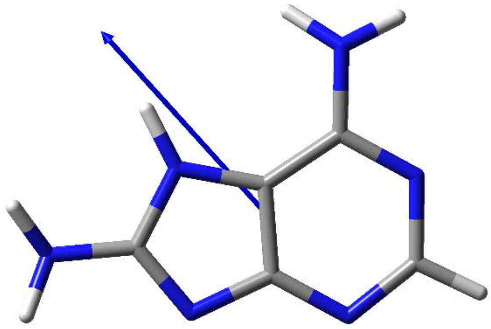

AD 7H C8-NH2 formamide

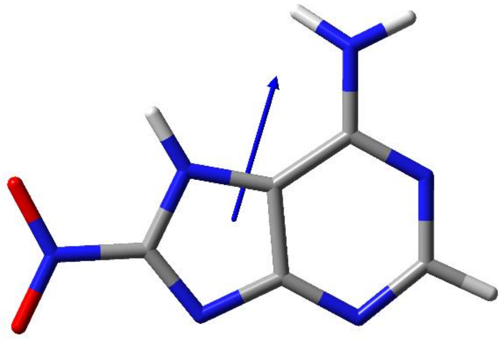

AD 7H C8-NO2 gas phase

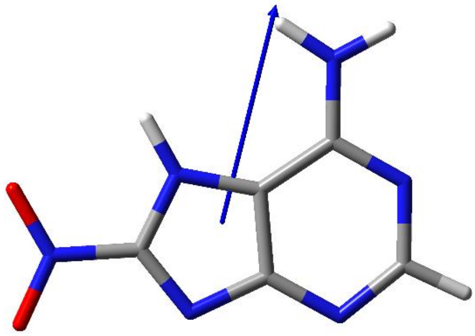

AD 7H C8-NO2 formamide

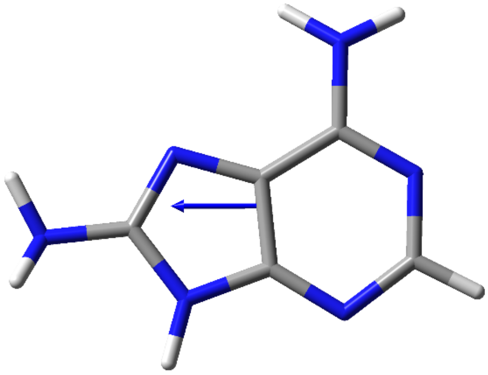

AD 9H C8-NH2 gas phase

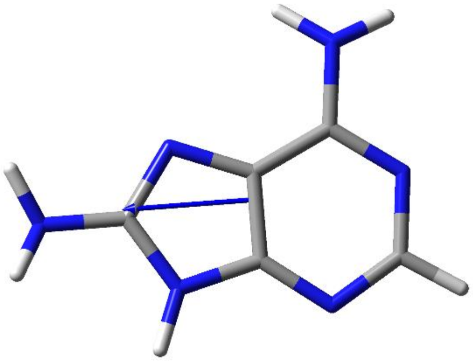

AD 9H C8-NH2 formamide

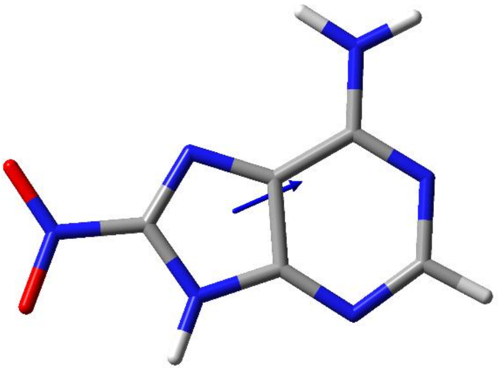

AD 9H C8-NO2 gas phase

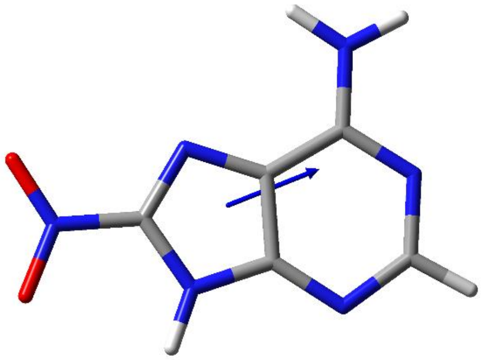

AD 9H C8-NO2 formamide

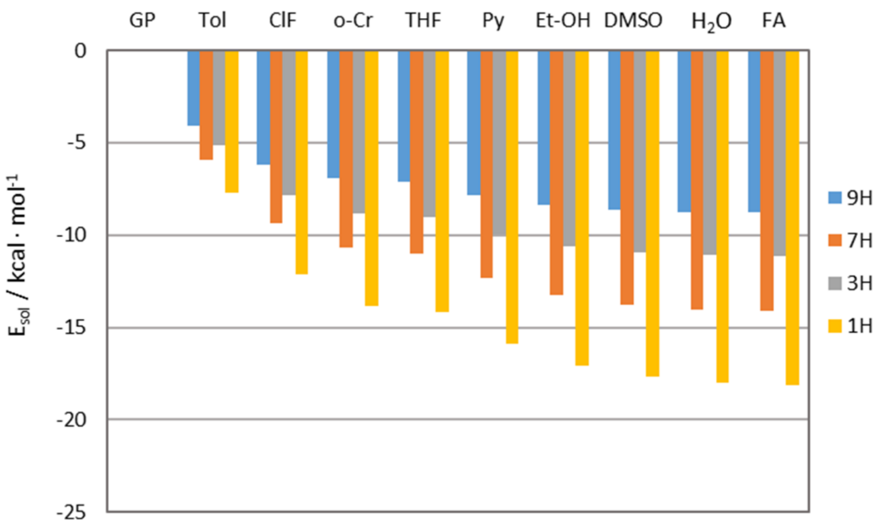
GP
Tol
ClF
o-Cr
THF
Py
Et-OH
DMSO
H2O
FA
0
-5
-10
-15
-20
-25
Esol / kcal · mol-1
9H
7H
3H
1H

(a)

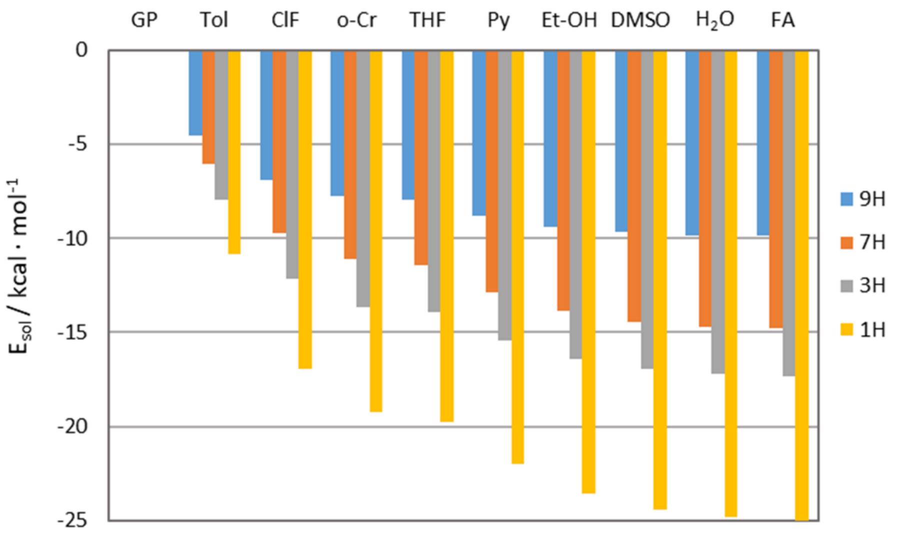
GP
Tol
ClF
o-Cr
THF
Py
Et-OH
DMSO
H2O
FA
0
-5
-10
-15
-20
-25
Esol / kcal · mol-1
9H
7H
3H
1H

(b)

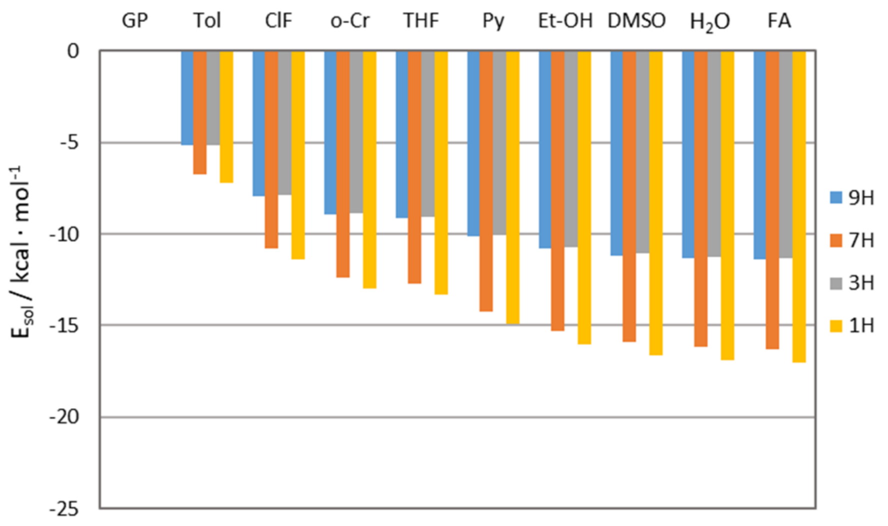
GP
Tol
ClF
o-Cr
THF
Py
Et-OH
DMSO
H2O
FA
0
-5
-10
-15
-20
-25
Esol / kcal · mol-1
9H
7H
3H
1H

(c)

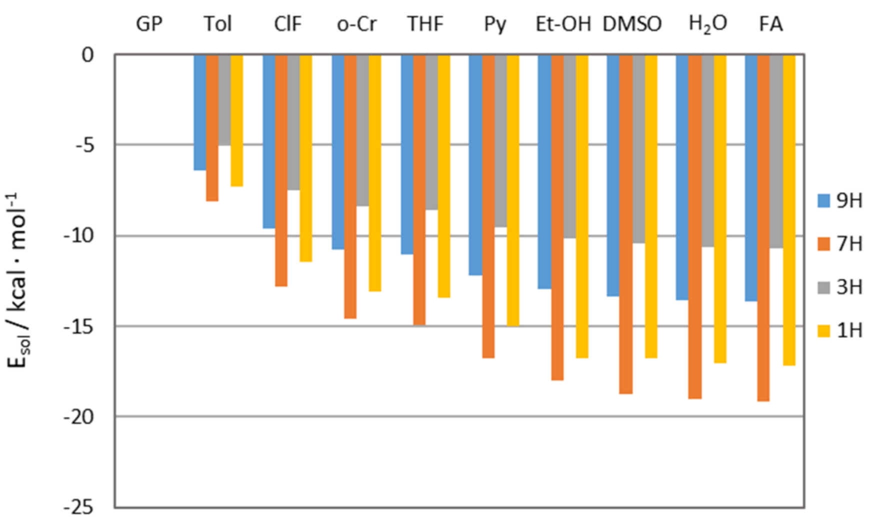

(d)

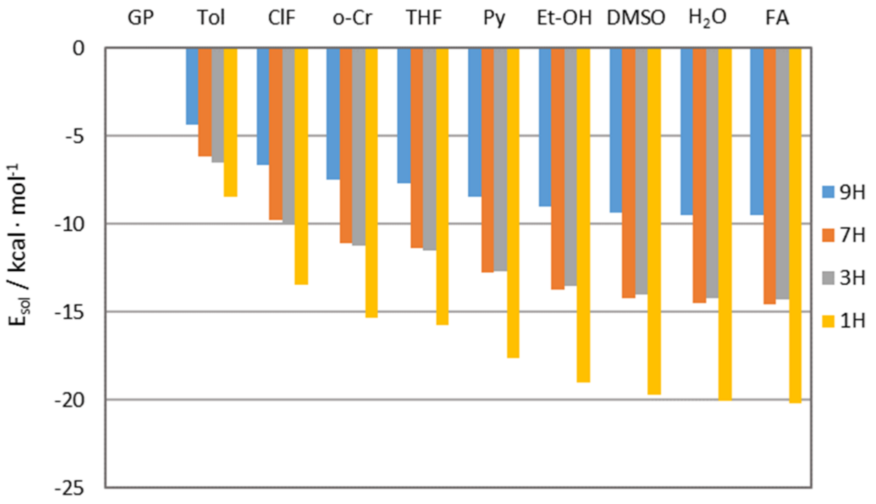

(e)

Figure S9. Solvation energies, E_{solv}, of unsubstituted (a), C8-NO_2 (b), C8-NH_2 (c), C2-NO_2 (d), and C2-NH_2 (e) substituted adenine tautomers in studied solvents.
